# Single-Cell Chromatin Accessibility Data Combined with GWAS Improves Detection of Relevant Cell Types in 59 Complex Phenotypes

**DOI:** 10.3390/ijms231911456

**Published:** 2022-09-28

**Authors:** Akash Chandra Das, Aidin Foroutan, Brian Qian, Nader Hosseini Naghavi, Kayvan Shabani, Parisa Shooshtari

**Affiliations:** 1 Department of Pathology and Laboratory Medicine, Western University, London, ON N6A 3K7, Canada; 2 Children’s Health Research Institute, Lawson Research Institute, London, ON N6C 2R5, Canada; 3 Department of Biosciences and Bioengineering, Indian Institute of Technology Guwahati, Guwahati 781039, India; 4 Department of Computer Science, Western University, London, ON N6A 5B7, Canada; 5 Ontario Institute for Cancer Research, Toronto, ON M5G 0A3, Canada

**Keywords:** open chromatin regions, GWAS, chromatin accessibility data, complex phenotypes

## Abstract

Several disease risk variants reside on non-coding regions of DNA, particularly in open chromatin regions of specific cell types. Identifying the cell types relevant to complex traits through the integration of chromatin accessibility data and genome-wide association studies (GWAS) data can help to elucidate the mechanisms of these traits. In this study, we created a collection of associations between the combinations of chromatin accessibility data (bulk and single-cell) with an array of 201 complex phenotypes. We integrated the GWAS data of these 201 phenotypes with bulk chromatin accessibility data from 137 cell types measured by DNase-I hypersensitive sequencing and found significant results (FDR adjusted *p*-value ≤ 0.05) for at least one cell type in 21 complex phenotypes, such as atopic dermatitis, Graves’ disease, and body mass index. With the integration of single-cell chromatin accessibility data measured by an assay for transposase-accessible chromatin with high-throughput sequencing (scATAC-seq), taken from 111 adult and 111 fetal cell types, the resolution of association was magnified, enabling the identification of further cell types. This resulted in the identification of significant correlations (FDR adjusted *p*-value ≤ 0.05) between 15 categories of single-cell subtypes and 59 phenotypes ranging from autoimmune diseases like Graves’ disease to cardiovascular traits like diastolic/systolic blood pressure.

## 1. Introduction

Many observable phenotypes in humans are referred to as complex phenotypes (traits/diseases) because multiple genes contribute to them either individually or through interactions with each other or the environment. Genome-wide association studies (GWAS) have been successful in uncovering thousands of genetic variants associated with complex phenotypes in humans [1]. Several studies have shown that GWAS variants of various complex phenotypes are preferentially located on non-coding regions of DNA and in particular on accessible chromatin sites [2,3,4]. Additionally, we and others have shown that the phenotype-relevant open chromatin sites are enriched in being accessible in specific cell types [2,5,6,7]. Examples include hepatocytes for low-density lipoprotein (LDL) [8], embryonic stem cells (H1-hESCs) for height [9], immune cells for autoimmune diseases [5,6], and cells of the nervous system for body mass index (BMI) [7]. For most complex phenotypes, the predicted relevant cell types are highly heterogeneous and the specific subsets of cells where phenotype-relevant regulatory sites are affected are largely unknown. Identifying specific subpopulations of cells underlying gene dysregulation of complex phenotypes, and in particular complex diseases, is crucial for understanding their biological mechanisms.

In previous studies, mostly the bulk sequencing technologies (e.g., DNase-I hypersensitive sequencing (DNase-I-seq) or the assay for transposase-accessible chromatin with high-throughput sequencing (ATAC-seq)) were used to measure chromatin accessibility. This provides an average chromatin accessibility profile across hundreds of thousands of cells. To fully understand epigenetic differences among cells and identify the specific subsets of cells underlying gene regulation in human complex phenotypes, it is necessary to study epigenetic modifications at the single-cell level. The single-cell sequencing technology is able to detect heterogeneity between individual cells and cell-specific changes that will lead to an overall higher “resolution” of the information collected from cell samples [10]. There are many cases where bulk sequencing methods lack the level of granularity required to sufficiently conserve data heterogeneity, such as with the complex tumor microenvironments in cancers and different cell compositions in various tissue types [11,12]. Single-cell sequencing leads to new cell subsets within known cell types, enabling the comprehensive characterization of cells from complex tissues [13].

Recently, several studies have integrated GWAS of complex phenotypes and single-cell RNA-seq (scRNA-seq) data to study the heterogeneity in the transcriptional profiles of individual cells as it relates to complex phenotypes [8,14,15,16]. However, these studies are not designed to capture epigenetic differences among cells and the functional consequences of these differences on gene regulation. The epigenetic heterogeneity among cells from the same cell type is highly informative, as it can reveal phenotype-relevant cell subsets defined by chromatin states that are not detectable by transcriptional analysis alone [17,18,19].

The technology of single-cell epigenomics has advanced rapidly [20,21,22], and this technology is now revolutionizing our understanding of gene regulation and continues to find many applications in the epigenetics of complex phenotypes. However, generating epigenomic data at the single-cell level is still quite expensive, and the single-cell epigenomic (e.g., scATAC-seq) data are not available for a wide range of human complex phenotypes. As an alternative option, publicly available GWAS data from complex phenotypes can be integrated with scATAC-seq databases containing samples from healthy individuals to predict the specific subsets of cells in which phenotype-relevant epigenetic changes have occurred.

Partitioning heritability using linkage disequilibrium score (LDSC) regression analysis has been the method of choice used by several studies to integrate GWAS data with epigenomic data at either the bulk or single-cell level [23]. LDSC regression analysis assesses the enrichment of phenotype-relevant genetic variants on the regulatory sites of various cell types and prioritizes phenotype-relevant cell types based on their enrichment levels. Peak data collected from bulk and single-cell chromatin accessibility datasets will provide the base pair locations of open chromatin regions, and combined with GWAS data, analysis can be performed exclusively on the risk variants found within these open chromatin regions. Different cell types have varying significance levels in association with a phenotype since open chromatin regions vary depending on the cell type itself. In other words, data across a multitude of cell types can be prepared with epigenomic functional categories to help understand the proportion of trait heritability explained exclusively by single nucleotide polymorphisms (SNPs) within open chromatin regions.

Here we have performed a large-scale study, where we used LDSC regression analysis to integrate the GWAS data of 201 human complex phenotypes with two large-scale chromatin accessibility databases in order to predict the relevant cell types in these traits. Our analysis highlights the strength of single-cell chromatin accessibility data combined with GWAS data, as it shows that there are phenotype-relevant cell subtypes that can be detected by the single-cell approach, while they might be missed when using bulk sequencing data.

## 2. Results

### 2.1. Integration of Bulk Chromatin Accessibility Data and GWAS Data

**Figure 1:**
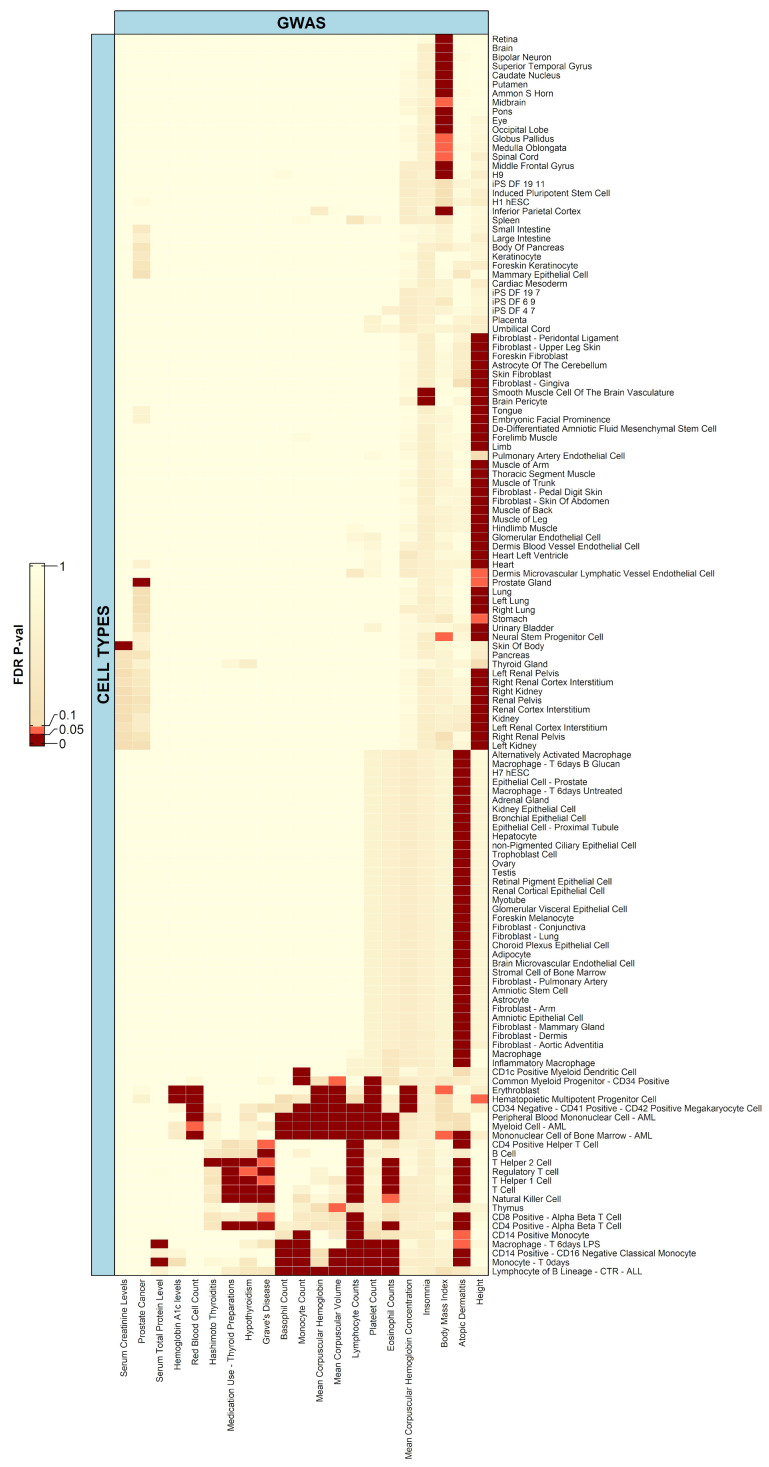
Heatmap depicting associations of 137 cell types form bulk chromatin accessibility data (from OCHROdb database) with 21 phenotypes. Of 201 phenotypes, 21 had a significant association (FDR adjusted *p*-value ≤ 0.05) with at least one cell type. In the heatmaps, dark red boxes represent significant association with an FDR adjusted *p*-value less than or equal to 0.05, while light red boxes represent trend association with an FDR adjusted *p*-value less than or equal to 0.10 but greater than 0.05.

For the bulk chromatin accessibility dataset obtained from OCHROdb, there were 21 phenotypes that had at least one significant association (false discovery rate (FDR) adjusted *p*-value ≤ 0.05) with any of the 137 cell types (Figure 1), an example of which is prostate cancer with one significant association with the cells of the prostate gland. Below we discuss some of these significant associations.

Atopic dermatitis (Eczema), an auto-immune disease, is a chronic inflammatory skin condition and like many inflammatory disorders, immune cells play a key role in it [24,25]. Atopic dermatitis showed significant associations with a number of cell types, including the T helper 1 cell, CD4-positive alpha–beta T cell, CD4-positive helper T cell, T cell, inflammatory macrophage, and the regulatory T cell, all of which are immune cells. Another broader category of cell types that were found to have a number of associations with atopic dermatitis were fibroblasts, and this association has been researched and confirmed previously [26].

Graves’ disease is an auto-immune disorder that results in the overproduction of thyroid hormones (hyperthyroidism), while Hashimoto’s thyroiditis is an auto-immune disorder in which the immune system attacks the thyroid tissue, resulting in the underproduction of thyroid hormones (hypothyroidism). Graves’ disease was significantly associated with immune cells like natural killer cells, the T cell, etc., whereas Hashimoto’s Thyroiditis was significantly associated with only the T helper 2 cell. The associations have been previously studied and well documented [27,28,29,30,31].

Body mass index or BMI, a polygenic trait and the most common proxy for obesity, was significantly associated with several cell types mainly from two organs, including the brain and eye. The associations signal the various regions of the brain which have been thoroughly researched and documented previously [32,33], and similarly with the eye [34]. The parts of the brain that play a role in BMI can be identified, however, as we only had cells from different regions of the whole brain and did not have brain cell types in OCHROdb, it was not possible to identify the specific subset of cells that play a role in that association.

Height is highly polygenic and has multiple cell types showing a significant association with it, and so has also been found by a study conducted by Guo et. al. [35]. Lymphocyte count [36] has significant associations with all the T lymphocytes, B lymphocytes, natural killer cells, and also with cell types that are a super-category of lymphocytes, such as the mononuclear cell of bone marrow, myeloid cells, peripheral blood mononuclear cells, and so on.

While many associations between the cell types and phenotypes are consistent with the discoveries from recent research, there were some associations that were unexpected to show a lack of significance. One example is the serum creatinine levels that have often been attributed to the functioning of the kidney [37]. Here, however, it only shows a significant association with cells of the skin of the body and none with renal or kidney cell types. It is important to note that a variety of dermatological diseases are more commonly seen in patients with renal transplants and chronic kidney disease (CKD) than the general population [38].

One hundred and eighty out of 201 phenotypes were not associated with any bulk cell type, among which many are autoimmune and cardiovascular diseases. We reasoned that this could be due to the lack of cell type resolution at the bulk sequencing level, and this motivated us to investigate the integration of GWAS data with single-cell chromatin accessibility data.

### 2.2. Integration of Single-Cell Chromatin Accessibility Data and GWAS Data

The associations that have been made between GWAS phenotypes and bulk chromatin accessibility data can be further magnified when single-cell chromatin accessibility data are used instead [39,40]. Switching from bulk to single-cell sequencing data provided two advantages. First, many such phenotypes which had no associations with bulk data showed up when integrated with single-cell datasets. Second, a greater resolution was produced as further unknown subtypes of cells were discovered to have significant associations with phenotypes which had been previously studied.

**Figure 2:**
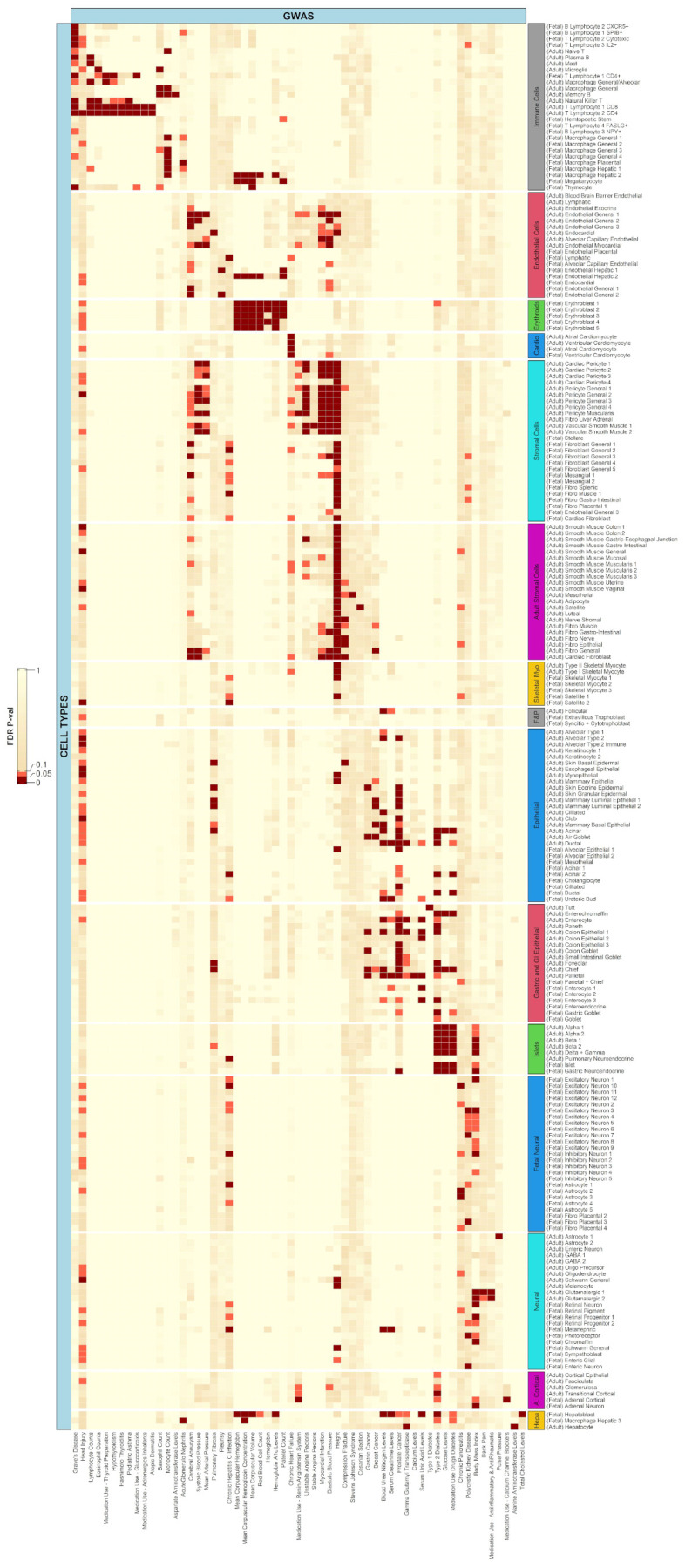
Heatmap depicting associations of scATAC-seq data for 222 cell types with 59 phenotypes. Of 201 phenotypes, 59 had a significant association (FDR adjusted *p*-value ≤ 0.05) with at least one cell type. The rows have been ordered according to different categories of cell types based on similarity and tissue composition. Here, cardio stands for cardiomyocytes, skeletal myo stands for skeletal myocytes, F&P stands for follicular and placental cells, a. cortical stands for adrenal cortical cells, and hepa stands for hepatocytes. In the heatmaps, dark red boxes represent significant association with an FDR adjusted *p*-value less than or equal to 0.05, while light red boxes represent trend association with an FDR adjusted *p*-value less than or equal to 0.10 but greater than 0.05.

From 21 phenotypes with a significantly associated cell type in the OCHROdb database, 19 had a significant cell type when integrated with the scATAC-seq too (Figure 2; Figures S1–S11). The missing two phenotypes were insomnia and serum total protein levels. The absence of insomnia can be explained as it was associated with brain pericyte and the smooth muscle cells of the brain vasculature, which were both absent in the scATAC-seq data, and so were any possible subtypes, from the single-cell database. The same can be said for the serum total protein levels. In the next sections, we highlight important associations between some cell types and phenotypes.

#### 2.2.1. Immune Cells

**Figure 3:**
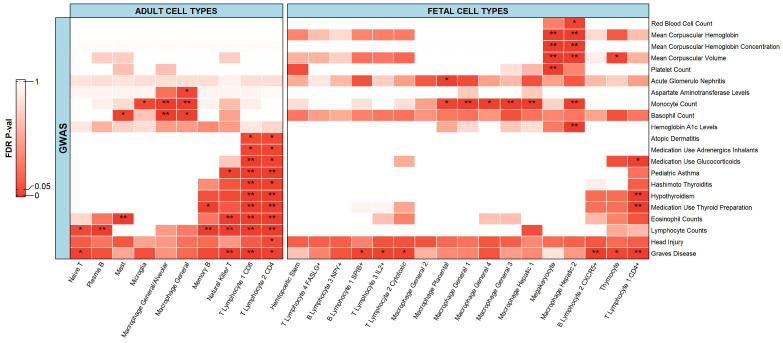
Heatmap depicting associations of immune cells from adult and fetal tissue cell types with respective phenotypes. The heatmap follows a gradient from red to white as the values go from 0.00 to 1.00. ** represents a significant association with an FDR adjusted *p*-value less than or equal to 0.01. * represents a significant association with an FDR adjusted *p*-value less than or equal to 0.05 but greater than 0.01.

We observed several significant associations (FDR adjusted *p*-value ≤ 0.05) for the immune cells when we integrated the GWAS datasets with the scATAC-seq data (Figure 3). More specifically, the immune cells were significantly associated with 21 phenotypes. Some significant associations were seen between single-cell data and phenotypes that can be matched intuitively as they are mostly some sort of metric (count/volume). Yet here we discuss the associations between single-cell data and important diseases such as Graves’ disease, hypothyroidism, Hashimoto’s thyroiditis, pediatric asthma, and atopic dermatitis. Adult cell types were associated with 14 phenotypes while fetal cell types were associated with 12 phenotypes. Examples of significant associations between adult cell types and diseases include Graves’ disease, hypothyroidism, Hashimoto’s thyroiditis, and pediatric asthma, and all of which were significantly associated with immune-related cells such as T lymphocyte 1 (CD8) and T lymphocyte 2 (CD4). Examples of significant associations for fetal cell types include Graves’ disease and hypothyroidism, and both were significantly associated with T lymphocyte 1 (CD4+). These associations have been previously studied and well documented [28,41,42]. For instance, Okajima et al., and Rydzewska et al. [28,42] highlighted the fact that autoimmune thyroid diseases, including Graves’ disease and Hashimoto’s thyroiditis, as well as hypothyroidism, which can be developed following Hashimoto’s thyroiditis disease, are characterized by intrathyroidal infiltration of the T lymphocytes CD4+ and CD8+, which are reactive to self-thyroid antigens. Associations between CD4 and CD8 T lymphocytes were also well document by Lloyd and Hessel [41]. Compared to the bulk results, within the single-cell results, more significant associations for Graves’ disease were observed.

With the single-cell results, the number of significant associations for atopic dermatitis with immune cell types were less than the bulk results and can be reasoned by comparing the type of immune cells. The single-cell dataset had more specific subtypes of immune cells, while the bulk dataset covered a broader class, and hence, it seems that atopic dermatitis had fairly more associations in the case of bulk analysis. However, it is important to note that in both the single-cell and bulk results, mainly T cells were significantly associated with atopic dermatitis. In addition, natural killer cells from bulk sequencing data had a significant association with atopic dermatitis, whereas natural killer T cells, a subtype of natural killer cells, had no significant association with atopic dermatitis. The autoimmune regulation between the two has been reviewed in detail by Seaman [43]. This suggests that natural killer T cells might not have a role in atopic dermatitis while other natural killer cell subtypes might.

#### 2.2.2. Islets and Neuroendocrine Cells

**Figure 4:**
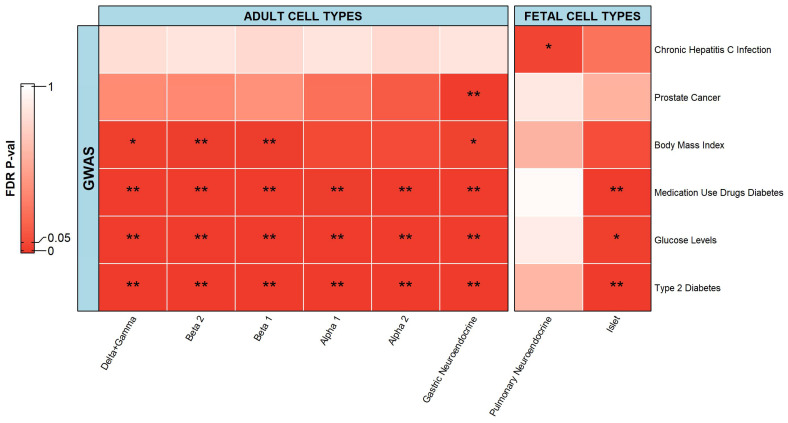
Heatmap depicting associations of islet and neuroendocrine cells from adult and fetal tissue cell types with respective phenotypes. The heatmap follows a gradient from red to white as the values go from 0.00 to 1.00. ** represents a significant association with an FDR adjusted *p*-value less than or equal to 0.01. * represents a significant association with an FDR adjusted *p*-value less than or equal to 0.05 but greater than 0.01.

For islets and neuroendocrine cells, there were six phenotypes that had a significant association with the scATAC-seq data (Figure 4). Adult cell types could be associated with five phenotypes while fetal cell types were associated with four phenotypes. There were significant associations between adult cell types and phenotypes including prostate cancer, type 2 diabetes, medication use drugs diabetes, and glucose levels, as well as BMI. In addition, there were significant associations between fetal pulmonary neuroendocrine cell types and chronic hepatitis C infection, as well as between islet cells and diabetic-related phenotypes including type 2 diabetes, medication use drugs diabetes, and glucose levels.

The pancreas contains clusters of cells that produce hormones. These clusters are known as pancreatic islets or islets of Langerhans which secrete hormones for controlling blood glucose levels. Alpha cells (secrete glucagon to raise the concentration of glucose in the bloodstream), beta cells (secrete insulin to inhibit glucose production and its level in the bloodstream), gamma cells (secrete pancreatic polypeptide to inhibit the release of glucagon in the bloodstream), and delta cells (secrete somatostatin to inhibit insulin, glucagon, and pancreatic polypeptide secretion in the bloodstream), are the four major types of cells present in the islets of Langerhans [44]. The role of islet cell types in the pathogenesis of diabetes, glucose levels, and insulin production has been thoroughly researched and documented [45], be it alpha cells [46,47], beta cells [48,49], or delta + gamma cells [50]. Genomic relations of islet cells with type 2 diabetes have also been studied via genome-wide association studies [51].

In this study, BMI was significantly associated with beta cells, delta + gamma cells, and gastric neuroendocrine cells. BMI is widely used to define obesity and as this progresses, can result in multiple disorders, including type 2 diabetes [52]. There exists a strong dogma asserting a relationship between beta cell mass and BMI [53]. Type 2 diabetes is associated with a reduced beta cell mass and function, thus causing inadequate insulin production [54]. BMI was not significantly associated with the pancreas in the OCHROdb analysis. This resolution could be achieved due to the specificity of the subtype of pancreatic cells. This observation highlights the advantage of using chromatin accessibility datasets at the single-cell level to detect relevant cell types.

In this study, gastric neuroendocrine cells were significantly associated with prostate cancer, type 2 diabetes, medication use drugs diabetes, and glucose levels, as well as BMI. Insulinomas are the most common types of neuroendocrine tumors (NETs), cancers that begin in specialized cells called neuroendocrine cells. These tumors secrete insulin, or less commonly proinsulin, leading to hypoglycemic (low blood glucose) symptoms, and relief is brought about with the administration of glucose. Thapi et al., showed a significant association between NETs and diabetes [55]. Lee et al., reported an association between gastric cancer and BMI, where compared with normal weight patients, underweight patients had a worse overall survival (OS) and disease-specific survival (DSS), whereas overweight and mildly to moderately obese patients had a better OS and DSS rate [56]. Therefore, apart from the association between gastric neuroendocrine cells and prostate cancer, where we could not find any related literature explaining this relationship, the other associations directly/indirectly are in agreement with the results of the mentioned studies.

The other finding here was the association between chronic hepatitis C infection and fetal pulmonary neuroendocrine cells, which has not been reported in any study so far. Bal et al., reported that patients having high doses of serum chronic hepatitis C RNA levels were 14.2 times more likely to show pulmonary dysfunction than non-viremic patients [57]. Segna and Dufour also reviewed the association between the hepatitis C virus (HCV) and endocrine and pulmonary manifestations [58]. Therefore, the fact that chronic hepatitis C infection can affect the lungs and other parts of the respiratory system has been well studied, but here we revealed an important finding at the cellular level, the main target cell type for chronic hepatitis C is the pulmonary neuroendocrine cell.

#### 2.2.3. Stromal Cells

**Figure 5:**
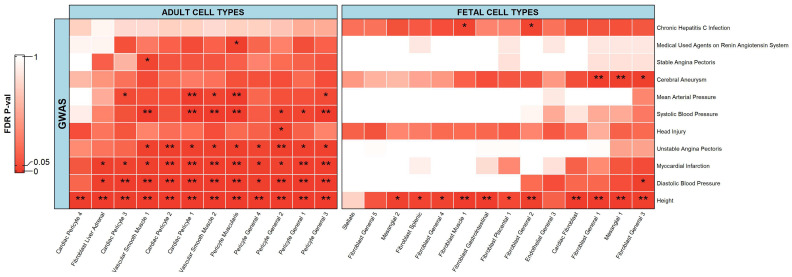
Heatmap depicting associations of stromal cells from adult and fetal tissue cell types with respective phenotypes. The heatmap follows a gradient from red to white as the values go from 0.00 to 1.00. ** represents a significant association with an FDR adjusted *p*-value less than or equal to 0.01. * represents a significant association with an FDR adjusted *p*-value less than or equal to 0.05 but greater than 0.01.

For the stromal cells, there were 11 phenotypes that had a significant association with the scATAC-seq data (Figure 5). Adult cell types were associated with nine phenotypes while fetal cell types were associated with four phenotypes. There were significant associations between adult cell types and phenotypes including height, head injury, and blood traits/diseases (such as diastolic blood pressure, systolic blood pressure, mean arterial pressure, myocardial infarction, unstable angina pectoris, stable angina pectoris, and medical used agents on renin–angiotensin system). There were also significant associations between fetal cell types and phenotypes including height, chronic hepatitis C infection, as well as blood related problems (such as diastolic blood pressure and a cerebral aneurysm).

In this study, significant associations were seen between adult/fetal cell types and blood traits/diseases. The proper delivery of blood into different parts of the body is essential for healthy tissue function. The anatomical substrate for this precise mechanism is the vascular unit, which is formed by endothelia cells, which form the inner lining of the blood vessel wall, as well as perivascular cells, referred to as pericytes, and vascular smooth muscle cells, which envelop the surface of the vascular tube [59,60]. Therefore, all these cell types are responsible for regulating capillary blood flow. Regarding stromal cell types, studies suggest that the loss of pericytes in vessels makes them hemorrhagic and hyper-dilated, thus affecting blood pressure, which leads to conditions such as diabetic retinopathy, edema, and embryonic lethality [60]. Špiranec et al., reported that endothelial C-type natriuretic peptide acts on pericytes for regulating blood flow and pressure and they highlighted the necessity of pericytes for the maintenance of normal microvascular resistance and blood pressure [61]. Touyz et al., and Michael et al., also reported that vascular smooth muscle cells have important roles in the regulation of blood pressure and defects in these cells could lead to several complications [62,63].

Unlike many significant associations between adult cell types and blood traits/diseases, fetal cell types were only associated with blood traits/diseases including a cerebral aneurysm and diastolic blood pressure. A cerebral aneurysm or brain aneurysm is a weak or thin spot on a brain artery that bulges out or balloons and fills with blood, which results in pressure being placed on the nerves or brain tissue [64,65]. This pressure may also result in the burst or rupture of a blood vessel, spilling blood into the surrounding tissue (called a hemorrhage), and can cause serious health issues such as a hemorrhagic stroke, brain damage, coma, and death [64,65]. Pediatric cerebral aneurysms are associated with a variety of disorders such as coarctation of the aorta, fibromuscular dysplasia, tuberous sclerosis, and polycystic kidney disease [66]. The latter may explain the relationship between cerebral aneurysms and fetal mesangial 1 cells, which are the stromal cells important for kidney glomerular homeostasis and the glomerular response to injury [67]. The few associations between fetal cell types and blood traits/diseases might be attributed to the general trend that the aberration in blood pressure occurs with aging due to structural changes in arteries [68].

Height is a highly polygenic trait on which extensive research has been done along with GWAS studies [35]. In this study, we saw significant associations between height and many adult/fetal stromal cell types. Zhang et al. [69] also produced the association between height GWAS data (smaller population) and single-cell chromatin accessibility data and generated similar results. Our study extends that conclusion to a larger population of multiple different ethnicities.

A significant association was observed between head injury and adult pericyte general 2 cell type. This association can be explained by the studies of Nakata et al., and Cai et al., where they reported that pericytes, with their multi-differentiation potency, are involved in neurogenesis after a transient ischemic stroke [70,71]. They also reported that pericytes could be used as a cell-based therapy after a stroke or other brain injuries to promote tissue restoration [70,71].

A significant association was seen between chronic hepatitis C infection and fibroblast cells including fibroblast muscle 1 and fibroblast general 2. This association might be explained by the fact that during liver injury, hepatic stellate cells are activated and undergo a transformation to proliferative, contractile myofibroblasts (muscle fibroblasts) [72,73]. This results in liver fibrosis, defined by the excessive accumulation of extracellular matrix proteins such as collagen, laminin, fibronectin, and elastin, and is currently considered as a wound healing response to chronic liver injury [72,73].

#### 2.2.4. Endothelial Cells

**Figure 6:**
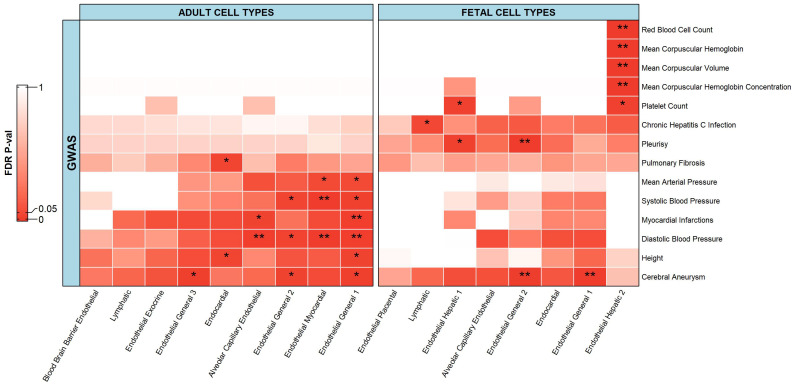
Heatmap depicting associations of endothelial cells from adult and fetal tissue cell types with respective phenotypes. The heatmap follows a gradient from red to white as the values go from 0.00 to 1.00. ** represents a significant association with an FDR adjusted *p*-value less than or equal to 0.01. * represents a significant association with an FDR adjusted *p*-value less than or equal to 0.05 but greater than 0.01.

In terms of endothelial cells, there were 14 phenotypes that had a significant association with the scATAC-seq data (Figure 6). Adult cell types could be associated with seven phenotypes while fetal cell types were associated with eight phenotypes. Adult endothelial cell types were significantly associated with pulmonary fibrosis, height, and blood related traits/diseases including a cerebral aneurysm, myocardial infarction, diastolic blood pressure, systolic blood pressure, and mean arterial pressure. Fetal endothelial cell types were also significantly associated with chronic hepatitis C infection, pleurisy, and blood related traits/diseases including cerebral aneurysm, platelet count, mean corpuscular hemoglobin, mean corpuscular hemoglobin concentration, mean corpuscular volume, and red blood cell count.

Almost all body tissues depend on a blood supply, and the blood supply depends on the endothelial cells, which form the internal linings of the blood vessels. These cells have roles including angiogenesis, hemostasis, and the regulation of vascular tone, as well as immune response via controlling the immune cell recruitment and extravasation into target tissues throughout the body [60,74]. Therefore, the significant associations between the fetal/adult endothelial cells and blood related traits/diseases seen here were expected, but here we showed exactly which subtypes of endothelial cell are linked to which type of blood related problems. Such an interesting detail seen here that was not reported previously was the association between fetal endothelial hepatic 2 cells and red blood cell phenotypes including mean corpuscular hemoglobin, mean corpuscular hemoglobin concentration, mean corpuscular volume, and red blood cell count.

In this study, chronic hepatitis C infection was significantly associated with lymphatic cells. Several studies have shown that the defects present in various lymphocyte populations in chronic hepatitis C patients and the extrahepatic diseases might be induced by a direct interaction between the chronic hepatitis C virus and lymphoid cells [75,76]. Many reports describing the existence of the chronic hepatitis C virus in B lymphocytes and B cell lymphoma, as well as in T lymphocytes and T cell lines, have been published [75,76]. These reports clearly validate our observed relationship between chronic hepatitis C infection and lymphatic cells.

We also observed significant associations between pleurisy and fetal endothelial cell types, including endothelial hepatic 1 and endothelial general 2. No study has directly linked the endothelial hepatic cell types with pleurisy, but a broader area in the direction has been researched, showing a pleural effusion in liver disease [77]. Pleurisy is a condition in which the two large, thin layers of tissue that separate your lungs from your chest wall, known as pleura, become inflamed, causing a sharp chest pain (pleuritic pain) that worsens during breathing [78]. Acute respiratory disorders such as pulmonary embolism, pneumonia, and pneumo-thorax are clinically significant conditions that may cause pleuritic pain [78]. Several studies have reported that endothelial cell damage has an important role in the pathogenesis of acute respiratory disorders and several biomarkers of endothelial damage have been tested in determining prognosis [79,80]. These findings validate the significant relationship between pleurisy and the fetal endothelial cell types observed in our study.

## 3. Discussion

The GWAS data provides invaluable information on the polygenic architecture behind complex traits and is most informative when combined with other genomic information in the downstream analyses. As of August 2022, there are over 5500 publications generating GWAS data recorded by the NHGRI-EBI Catalog alone; [1] therefore, the field of association studies has become quite expansive ever since the first GWAS was published in 2005 [81]. While GWAS provides important information regarding the association of genetic variants with phenotypes of interest, the GWAS data type alone is not sufficient to unravel the specific cellular and molecular mechanisms underlying complex phenotypes. In this study, we have integrated GWAS and chromatin accessibility data to identify the specific cell types in which phenotype-relevant epigenetic changes are likely to happen. Here, we provided the most optimal and informative conditions when performing partitioning heritability analysis through GWAS integration, by not only accounting for linkage disequilibrium but taking advantage of the heterogeneity of bulk and single-cell chromatin accessibility sequencing data. Compared to previous studies, here we scaled up our analysis and generated a large collection of associations between a wide array of cell types at the bulk and single-cell chromatin accessibility level and tens of GWAS phenotypes.

Bulk sequencing refers to the sequencing of DNA with every sample containing thousands of cells. In turn, the data available in every sample are an average of the data across many cells, and in the case of open chromatin regions, the average level of accessibility across a large number of cells in a sample is represented. At a population level, bulk sequencing is able to provide an effective overview of chromatin accessibility patterns; however, it does have its limitations in the preservation of data heterogeneity and overall accuracy. It cannot provide insight into the variable levels of accessibility between individual cell subtypes [82]. Signals that are less frequently seen within a population of cells are drowned out, so the data is lost. In contrast, the single-cell sequencing technology is able to detect heterogeneity between individual cells and cell-specific changes that will lead to an overall higher “resolution” of information collected from cell samples [10]. Ultimately, single-cell sequencing will lead to new cell subsets within known cell types, enabling the comprehensive characterization of cells from complex tissues [13]. In this study, we integrated the GWAS data of 201 phenotypes (diseases, biomarkers, and medication usage) with bulk chromatin accessibility data measured by DNase-I-seq and found significant results for at least one cell type in 21 complex phenotypes, such as strong associations with atopic dermatitis, Graves’ disease, BMI, and height. With the integration of single-cell chromatin accessibility data measured by scATAC-seq and taken from 222 cell types (111 adult and 111 fetal cell types), the resolution of association was magnified, enabling the identification of further cell types. At the single-cell level, significant correlations were found between 15 categories of cell types (222 cell types) and 59 phenotypes ranging from autoimmune diseases like Graves’ disease to cardiovascular traits like diastolic/systolic blood pressure. For those phenotypes with no significantly identified cell types (neither at the bulk nor single-cell resolution), the lack of association may be caused by the relatively lower sample sizes of their corresponding GWAS study (Table S1).

We pointed out many of the generated associations in the literature that was already thoroughly documented, such as the associations made between immune cells with Graves’ disease, hypothyroidism, and hyperthyroidism. However, in many cases, the more uncommon associations are not sufficiently considered in practice or research. It was often the case that little to no literature existed to support some of the significant associations generated from this paper, but we tried to speculate the results by addressing the association from the literature between phenotype and a broader category of cell type (e.g., tissue), instead of a particular subtype of cell. The results uncovered in this study opened the possibility of researching the mechanism of these complex phenotypes through the various cell subtypes that were associated with them. While the results of this study branch off towards research outlining well-known mechanisms of disease pathogenesis, there is also an opportunity to open new and potential research avenues for discovering cell-type specific pathways not yet considered when studying the development of diseases. Associations between cell types and phenotypes from this study can shed light upon future under-researched areas of diseases, medicines, and other traits.

In our study, we have considered chromatin accessibility data (bulk and single-cell) as they provide important associations between phenotypes and particular cell types based on the open chromatin regions within the genome. However, the GWAS data [83] can also be integrated with other sequencing data types such as RNA-seq [84,85,86] or DNA methylation [87,88] data to draw meaningful insights into multiple phenotypes. Each of these data types provide important information about the gene regulation genome-wide. RNA-seq provides information about the genes that are being expressed [89], ATAC-seq or DNase-I hypersensitive sequencing provides information about the potentially active gene switches and transcription factor-binding sites [90], and DNA methylation provides information regarding gene regulation and transcriptional activities [91]. Therefore, combining these data will reveal a more comprehensive view of gene regulation. While both scRNA-seq and methylation can be used to verify our results, there might be slight variations owing to the difference in the basic information these sequencing techniques capture.

## 4. Materials and Methods

### 4.1. GWAS Data

GWAS studies identify and associate phenotypes to variations in the genome. GWAS intends to identify and map the polygenic architecture of phenotypes through SNP alleles that exist in a significantly different proportion for a specific trait group (e.g., patients having a specific disease) when compared with controls (e.g., unaffected patients).

There is a plethora of publicly available GWAS datasets for different phenotypes. For this study, we have used a cross-population atlas of such associations with around 201 phenotypes taken from a study by Sakaue et al. [92]. The paper contains GWAS data for 220 complex phenotypes (diseases, biomarkers, and medication usage) from BioBank Japan (BBJ), UK Biobank (UKB), and FinnGen (n_total_ = 628,000) and identified 5343 new loci, substantially improving the resolution of genomic mapping of human phenotypes, while also diversifying the database by incorporating varying ethnicities. Of these, 201 were selected for this study, eliminating the inaccessible, missing, or repeated ones.

### 4.2. Chromatin Accessibility Data

The choice of datasets, starting with bulk chromatin accessibility data to single-cell chromatin accessibility data, was made to identify the resolution that can be produced as one goes from analyzing bulk sequencing to single-cell sequencing. Both selected bulk and single-cell chromatin accessibility datasets broadly scan over the several human tissue cell types and they are not focused on only certain organs which would have limited the scope of our study. We obtained the bulk chromatin accessibility data from our previously built database called OCHROdb (https://dhs.ccm.sickkids.ca/ accessed on 27 May 2022) [5,93]. OCHROdb is one of the largest bulk chromatin accessibility databases available publicly that contains a diverse range of cell types and tissues. OCHROdb was originally generated by integrating sequencing-based open chromatin data from 828 samples generated by four international consortia (ENCODE, Roadmap, Blueprint, and NIH GGR). The samples were uniformly processed and quality checked to ensure the open chromatin sites pass the replication test. OCHROdb comprises ~1.5 million peaks (open chromatin regions) across 194 cell types and cell lines. Fifty-seven/one hundred and ninety four cell types/cell lines were not categorized as normal, healthy cells, and were therefore not considered in this analysis. We prepared peak files for each of the remaining 137 cell types in the BED format, which contains data on the start and end positions of open chromatin regions along with the chromosome number. To accommodate for flanking regions, 100 base pairs have been added to each side of every peak region location.

**Figure 7:**
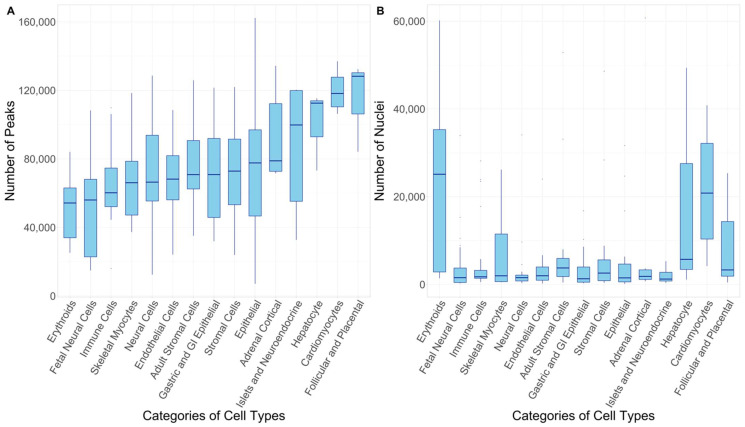
(**A**) Box plot depicting the number of peaks per category of cell type to visualize the number of open chromatin regions. The boxes have been sorted in increasing order of median. (**B**) Box plot depicting the number of nuclei per category of cell type to visualize the resolution of the study. The boxes are in the same order as section A. Details regarding all the particular cell type have been provided in the Table S2.

For the scATAC-seq data, we chose a dataset generated by Zhang et al., (Figure 7, Table S2) due to the availability of a wide range of cell types both from adult and fetal tissues in this dataset [69]. This dataset contains scATAC-seq data for 30 adult human tissue types which was integrated with previously available scATAC-seq data of 15 fetal tissue types [94] to reveal the status of open chromatin for ~1.2 million candidate cis-regulatory elements (cCREs) in 222 distinct cell types comprised of >1.3 million nuclei. The genome build was hg38 for adult cell types, while it was hg19 for fetal cell types, so we used the UCSC LiftOver tool to lift hg19 to hg38 [95]. There were no unhealthy cell types or cell lines, so all of the cell types were used in our analysis. Once retrieved, the 222 cell types were divided into 2 batches of 111 adult and 111 fetal cell types. We prepared peak files for each of the 222 cell types in the BED format. The peak files (“.bed” format) obtained from the dataset already included the flanking sequence of 100 base pairs. Zhang et al., also integrated these 222 cell types with the GWAS data for multiple phenotypes, obtained from the study of Buniello et al. [1]. However, the dataset used in our study is newer and more comprehensive, capturing sufficient diversity in the population owing to the meta-analyses of independent biobanks and the scope of phenotypes. Our study not only contains disease endpoints but also traits such as blood cell count, diastolic/systolic blood pressure, and medication usage associations [96]. Our selected GWAS dataset also consists of around 108 phenotypes on which GWAS has never been conducted in the East Asian populations.

We first integrated the GWAS datasets with the bulk chromatin accessibility data and then with single-cell chromatin accessibility data, enabling us to identify further unknown cell subtypes and enhance the resolution of the associations. Table S1 contains information about the phenotypes including the number of cases, controls, and percentage of cases per control. The 201 phenotypes include 143 diseases, 22 medication usage phenotypes, and 36 other traits.

### 4.3. Prioritizing Cell Types Using Linkage Disequilibrium Score Regression Analysis

Although there is a vast number of published GWAS available online, and thousands of loci associated with complex phenotypes have already been mapped through these numerous studies, there are a few outstanding complications with these associations that must be considered before moving forward with the data integration. Linkage disequilibrium (LD) is one of the major complications with downstream analysis using GWAS data [97]. There is an existing correlation between alleles in the genome for many reasons, including allele proximity on the chromosome, mutation, genetic drift, and other confounding factors [97]. One of the main reasons is due to crossing over during meiosis. During this process, some regions of the genome are more likely to stay together than others. Therefore, for a sample disease phenotype, the causal variant may be present firmly in a large population. Still, it would be difficult to isolate and identify it as non-causal variants linked to the causal variant would also be simultaneously present in many positive cases and absent in many controls. In other words, non-causal SNPs in LD with a causal SNP will have inflated levels of association with a potential disease or the trait of interest.

We used a tool called LDSC (version 1.0.1) to account for this complication, by distinguishing between inflated test statistics from LD and other confounding biases found in statistical genetics [23,98]. The LDSC utilizes a stratified LD score regression and estimates the variance explained by all the SNPs on a chromosome when testing the association of a particular SNP to a phenotype. LDSC analysis is specifically designed for finding out how partitioned heritability can be explained by the risk variants that are located in specific genomic regions, which in this case, refers to open chromatin regions. It ultimately employs a powerful and accurate correction factor, refining association data to show a true, unconfounded polygenic signal.

We integrated the GWAS of 201 phenotypes with the chromatin accessibility data (both bulk and single-cell) using LDSC-based partitioning heritability analysis, and identified specific cell types with the significant enrichment of phenotype-relevant variants (e.g., SNPs) on their chromatin accessibility sites. Our core partitioning heritability analysis workflow can be summarized in the following steps: the preparation of peak data from chromatin accessibility databases, LD score regression calculation, GWAS integration, and data visualization.

In our study, we used chromatin accessibility datasets both at the bulk and single-cell level, and, as mentioned earlier, prepared their peaks (i.e., accessible chromatin sites) information across various cell types and tissues in the BED file format.

The next step in the partitioned heritability workflow was to calculate the LD scores for each SNP found within the open chromatin regions. To do so, firstly, for each of the cell types, the “.bed” file was used along with “.bim” PLINK files, containing information on known SNPs, to generate binary annotation files for each cell type and chromosomes 1–22. This was done through the “make_annot.py” script provided by the LDSC toolkit from the LDSC github page (https://github.com/bulik/ldsc accessed on 27 May 2022). At this stage, for every cell type, SNPs that are found within the inputted open chromatin regions were listed in the binary annotation files as 1s and the ones that are absent, as 0s. Then the “ldsc.py” script from the LDSC github page was used to calculate the LD scores for each SNP found within the open chromatin regions. This required the input of the binary annotation files, as well as the “.bim” PLINK file. We used the HapMap3 SNP data as a checklist of qualifying SNPs to include in the LD calculation. The resulting output files contained LD score information for every qualifying SNP.

The next step was the integration of the GWAS data with the chromatin accessibility data using the “ldsc.py” script from the LDSC github page. Here we used the GWAS summary statistic files in conjunction with the generated LD files to create links between cell types and phenotype SNPs using the LDSC toolkit. This integration step was repeated for all of the different phenotypes to be considered in the analysis. For each association between phenotypes and cell types, a coefficient *p*-value was calculated, signifying the potential relevance of each cell type to the specific phenotype. Thus, a significant *p*-value represented a significant contribution of the open chromatin sites of a cell type to SNP heritability for the specific phenotype. To correct for multiple testing, an FDR with a threshold of ≤0.05 was used to adjust the *p*-values within each cell type batch, that is, bulk, adult single-cell, and fetal single-cell [99].

Once the resulting *p*-values were adjusted, heatmaps were constructed to effectively visualize the data and derive reasonable conclusions from it. To generate the heatmaps, the ComplexHeatmap R package (version 2.10.0) [100] was used. First and foremost, the results from the bulk chromatin accessibility data (OCHROdb) were generated into heatmaps. Only those phenotypes were selected which had at least one cell type with an FDR adjusted *p*-value ≤ 0.05. A similar process was then repeated on the scATAC-seq datasets, resulting in 222 cell types from both adult and fetal cell types. For a more comprehensive understanding of the associations in the case of scATAC-seq data, the cell types were divided into 15 categories, based on their similarity and tissue composition which was loosely based on the Zhang et al., study (Table 1). Heatmaps were generated for each category that further subdivided the fetal and adult cell types along the *X*-axis, while the *Y*-axis contained the GWAS phenotypes that had at least one significant (≤0.05) cell type in the particular category.

## 5. Conclusions

For this study, we integrated the GWAS data of 201 complex phenotypes (diseases, biomarkers, and medication usage) with bulk and single-cell chromatin accessibility sequencing data to uncover those cell types that are significantly associated with these com-plex phenotypes. First, we integrated the GWAS data of these 201 phenotypes with bulk chromatin accessibility sequencing data and found significant results for at least one cell type in 21 complex phenotypes. Then we integrated the GWAS data of these 201 phenotypes with chromatin accessibility sequencing data at the single-cell level and found significant associations between 15 categories of cell types and 59 phenotypes. Therefore, the resolution of association was magnified at the single-cell level enabling the identification of further cell types, compared to the bulk analysis results. The associations between cell types and phenotypes from this study can shed light upon future under-researched areas of diseases, medicines, and other traits. Further studies should be done on the identified subsets of cells that contribute to each phenotype to understand gene regulatory mechanisms of a given phenotype. The identified significant associations can be linked with several works of literature that investigate biological mechanisms behind disease pathogenesis.

## Figures and Tables

**Table 1:** Categories of cell types along with number of adult and fetal cell types.

Category of Cell Types	Number of Adult Cell Types	Number of Fetal Cell Types
Immune Cells	10	17
Endothelial Cells	9	8
Erythroids	0	5
Cardiomyocytes	2	2
Stromal Cells	12	14
Adult Stromal Cells	22	0
Skeletal Myocyte	2	5
Follicular and Placental	1	2
Epithelial	19	9
Gastric and GI Epithelial	12	7
Islets and Neuroendocrine	6	2
Fetal Neural Cells	0	25
Neural Cells	11	11
Adrenal Cortical Cells	4	2
Hepatocytes	2	1

## Data Availability

All the data, source codes, and generated results are available in the GitHub repository named as ochroGWAS (https://github.com/shooshtarilab/ochroGWAS accessed on 27 May 2022).

## Supplementary Materials

The following supporting information can be downloaded at: https://www.mdpi.com/article/10.3390/ijms231911456/s1.
