# Supplementary material for: Single-Cell Chromatin Accessibility Data Combined with GWAS Improves Detection of Relevant Cell Types in 59 Complex Phenotypes
Source: Int J Mol Sci. 2022 Sep 28;23(19):11456. doi: 10.3390/ijms231911456 (PMC9570273; doi:10.3390/ijms231911456)
Supplement: Supplementary file 1 [file ijms-23-11456-s001.zip › Supplementary Figures.pdf]

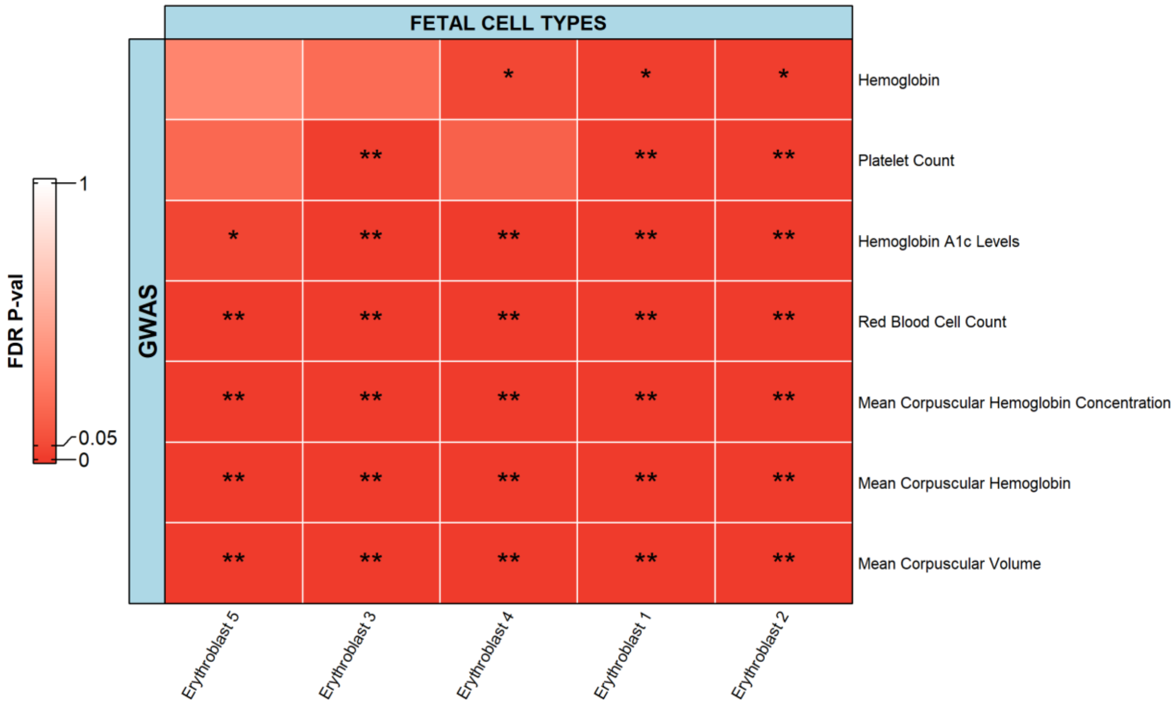


**Figure S1.** Heatmap depicting associations of erythroids from fetal tissue cell types with respective phenotypes. The heatmap follows a gradient from red to white as the values go from 0.00 to 1.00. ** represents a significant association with an FDR adjusted p-value less than or equal to 0.01. * represents a significant association with an FDR adjusted p-value less than or equal to 0.05 but greater than 0.01.

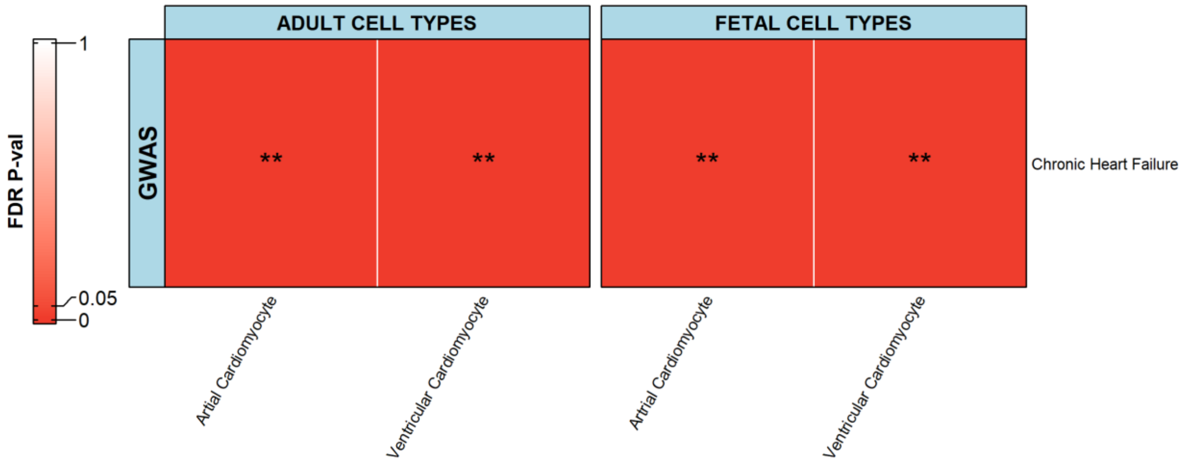


**Figure S2.** Heatmap depicting associations of cardiomyocytes from adult and fetal tissue cell types with respective phenotypes. The heatmap follows a gradient from red to white as the values go from 0.00 to 1.00. ** represents a significant association with an FDR adjusted p-value less than or equal to 0.01. * represents a significant association with an FDR adjusted p-value less than or equal to 0.05 but greater than 0.01.

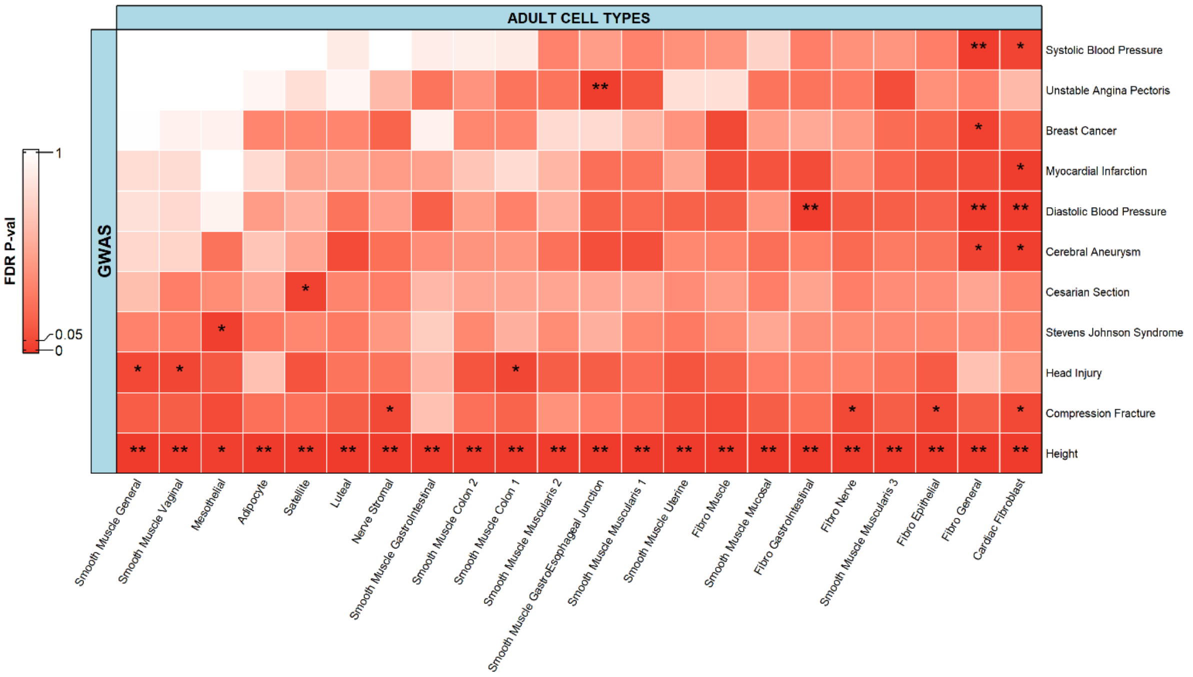


**Figure S3.** Heatmap depicting associations of adult stromal cells from adult tissue cell types with respective phenotypes. The heatmap follows a gradient from red to white as the values go from 0.00 to 1.00. ** represents a significant association with an FDR adjusted p-value less than or equal to 0.01. * represents a significant association with an FDR adjusted p-value less than or equal to 0.05 but greater than 0.01.

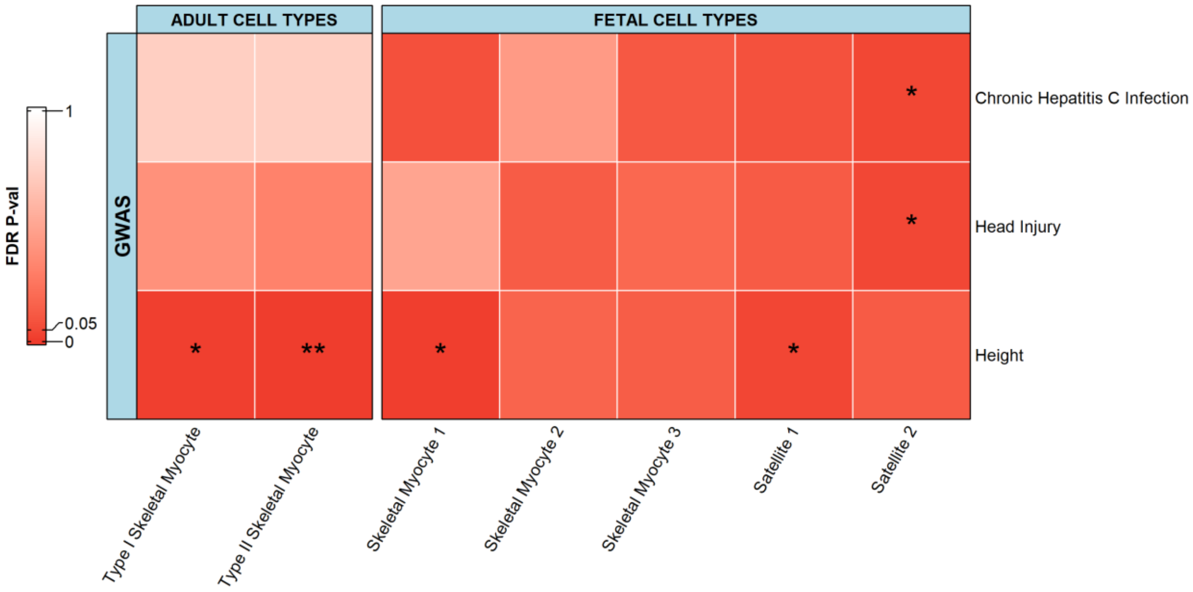


**Figure S4.** Heatmap depicting associations of skeletal myocytes from adult and fetal tissue cell types with respective phenotypes. The heatmap follows a gradient from red to white as the values go from 0.00 to 1.00. ** represents a significant association with an FDR adjusted p-value less than or equal to 0.01. * represents a significant association with an FDR adjusted p-value less than or equal to 0.05 but greater than 0.01.

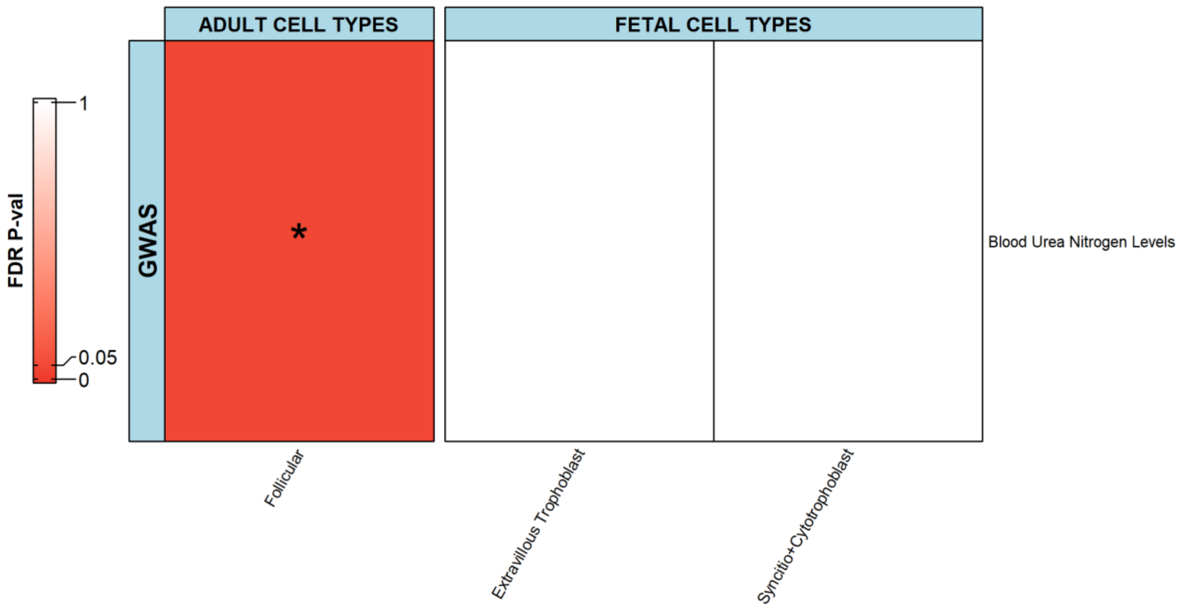


**Figure S5.** Heatmap depicting associations of follicular and placental cells from adult and fetal tissue cell types with respective phenotypes. The heatmap follows a gradient from red to white as the values go from 0.00 to 1.00. ** represents a significant association with an FDR adjusted p-value less than or equal to 0.01. * represents a significant association with an FDR adjusted p-value less than or equal to 0.05 but greater than 0.01.

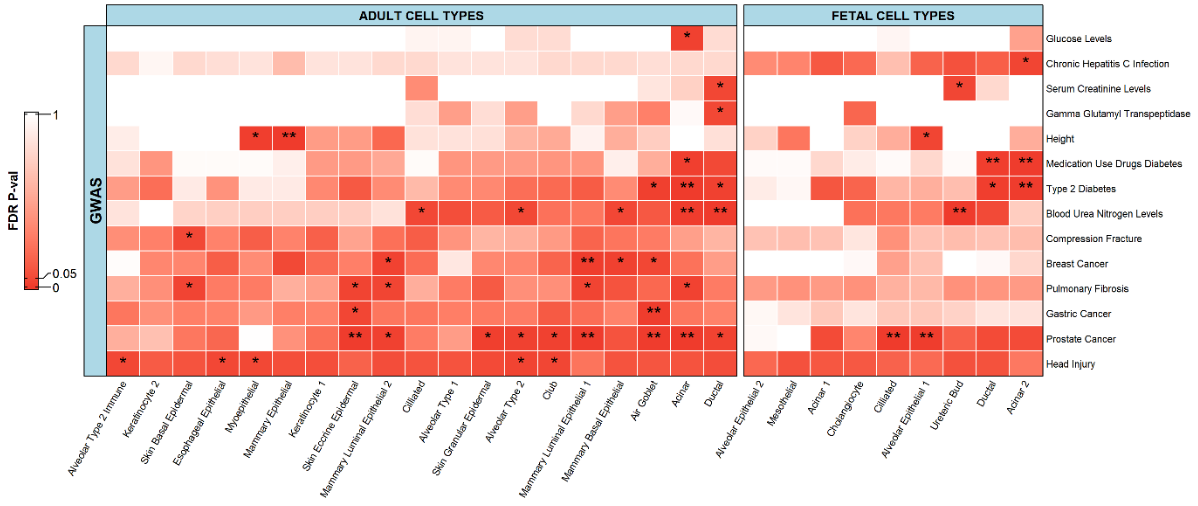


**Figure S6.** Heatmap depicting associations of epithelial cells from adult and fetal tissue cell types with respective phenotypes. The heatmap follows a gradient from red to white as the values go from 0.00 to 1.00. ** represents a significant association with an FDR adjusted p-value less than or equal to 0.01. * represents a significant association with an FDR adjusted p-value less than or equal to 0.05 but greater than 0.01.

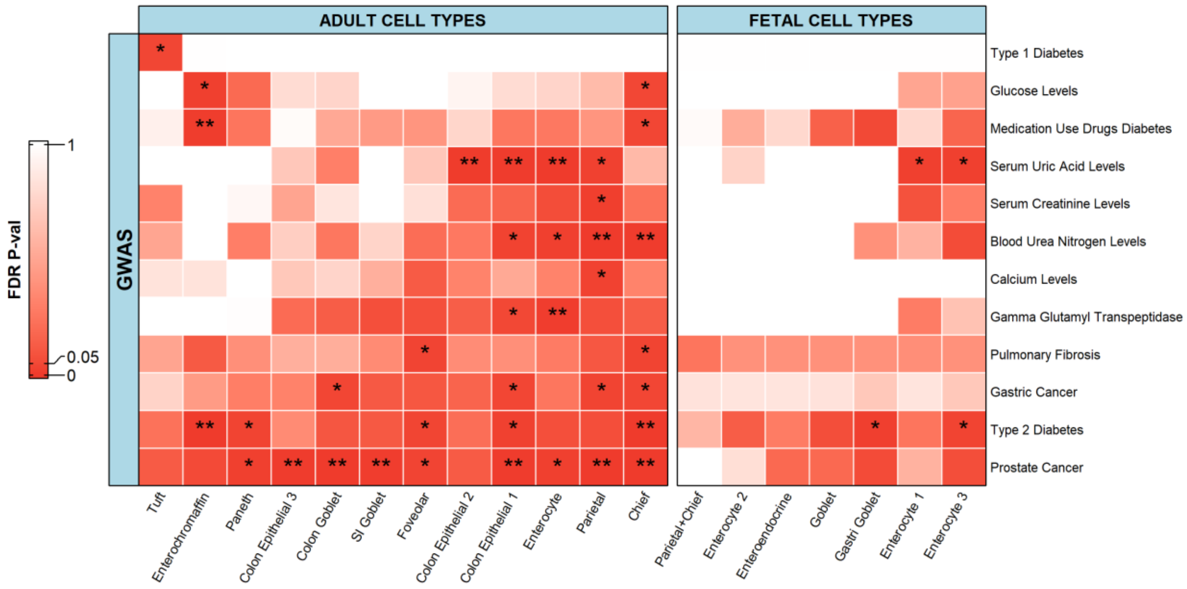


**Figure S7.** Heatmap depicting associations of gastric and gastro-intestinal epithelial cells from adult and fetal tissue cell types with respective phenotypes. The heatmap follows a gradient from red to white as the values go from 0.00 to 1.00. ** represents a significant association with an FDR adjusted p-value less than or equal to 0.01. * represents a significant association with an FDR adjusted p-value less than or equal to 0.05 but greater than 0.01.

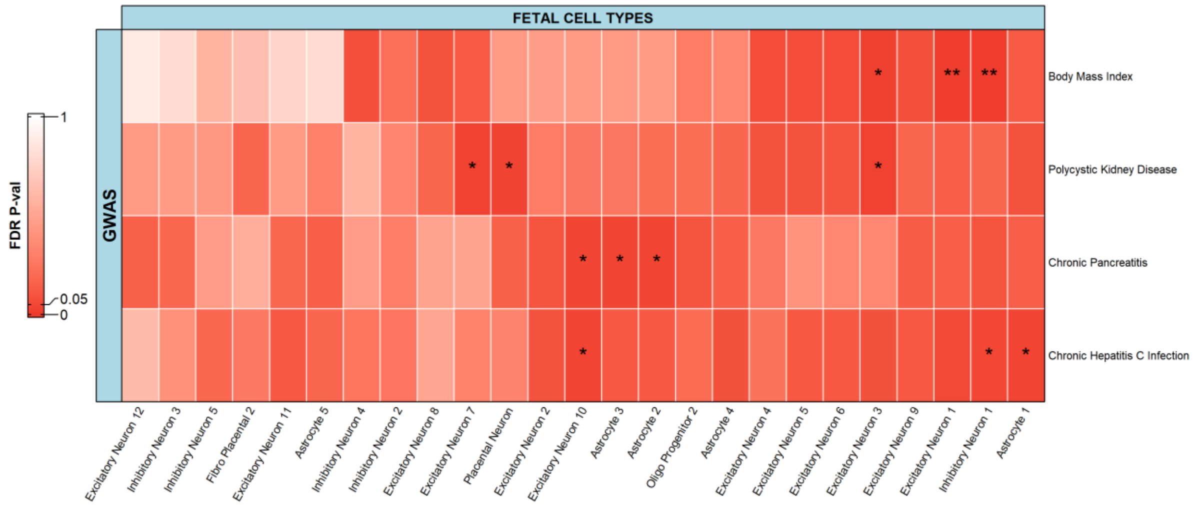


**Figure S8.** Heatmap depicting associations of fetal neural cells from fetal tissue cell types with respective phenotypes. The heatmap follows a gradient from red to white as the values go from 0.00 to 1.00. ** represents a significant association with an FDR adjusted p-value less than or equal to 0.01. * represents a significant association with an FDR adjusted p-value less than or equal to 0.05 but greater than 0.01.

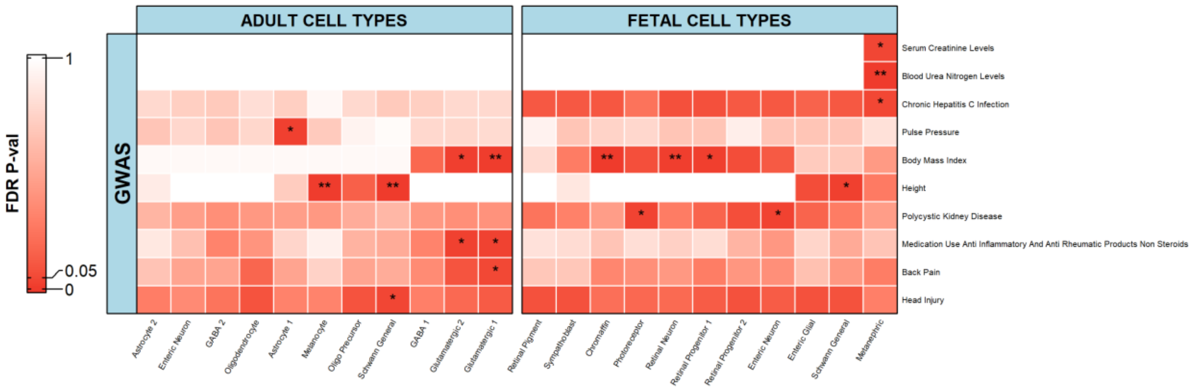


**Figure S9.** Heatmap depicting associations of neural cells from adult and fetal tissue cell types with respective phenotypes. The heatmap follows a gradient from red to white as the values go from 0.00 to 1.00. ** represents a significant association with an FDR adjusted p-value less than or equal to 0.01. * represents a significant association with an FDR adjusted p-value less than or equal to 0.05 but greater than 0.01.

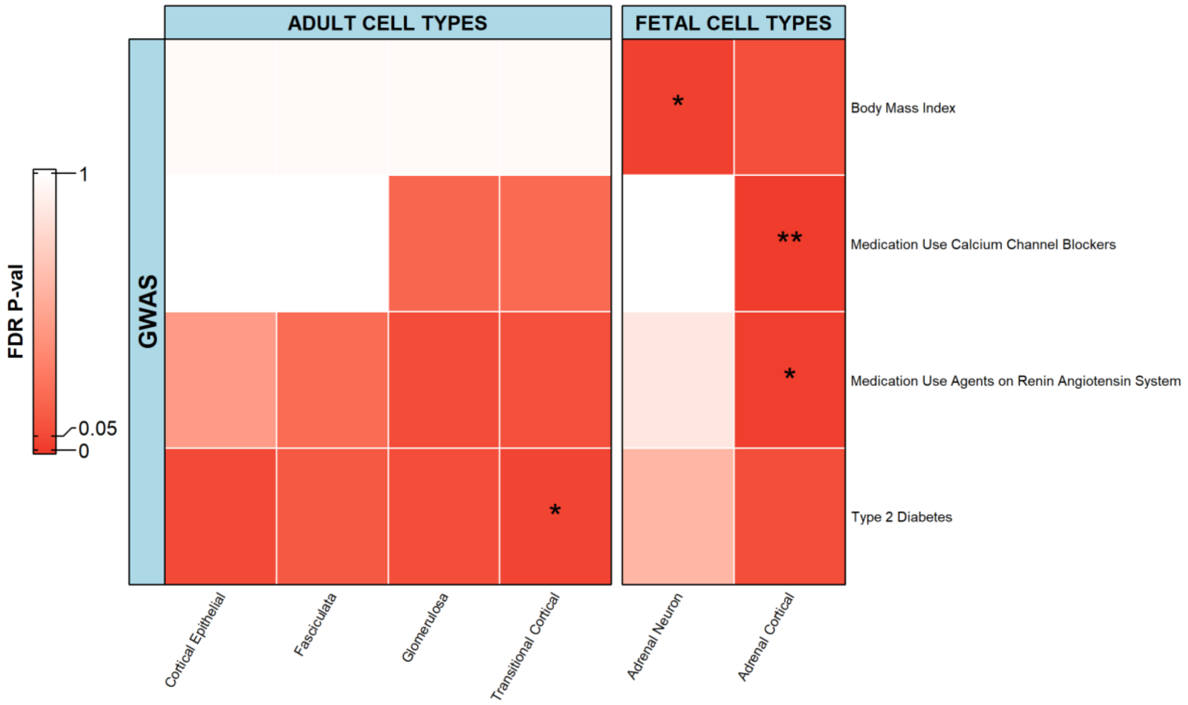


**Figure S10.** Heatmap depicting associations of adrenal cortical cells from adult and fetal tissue cell types with respective phenotypes. The heatmap follows a gradient from red to white as the values go from 0.00 to 1.00. ** represents a significant association with an FDR adjusted p-value less than or equal to 0.01. * represents a significant association with an FDR adjusted p-value less than or equal to 0.05 but greater than 0.01.

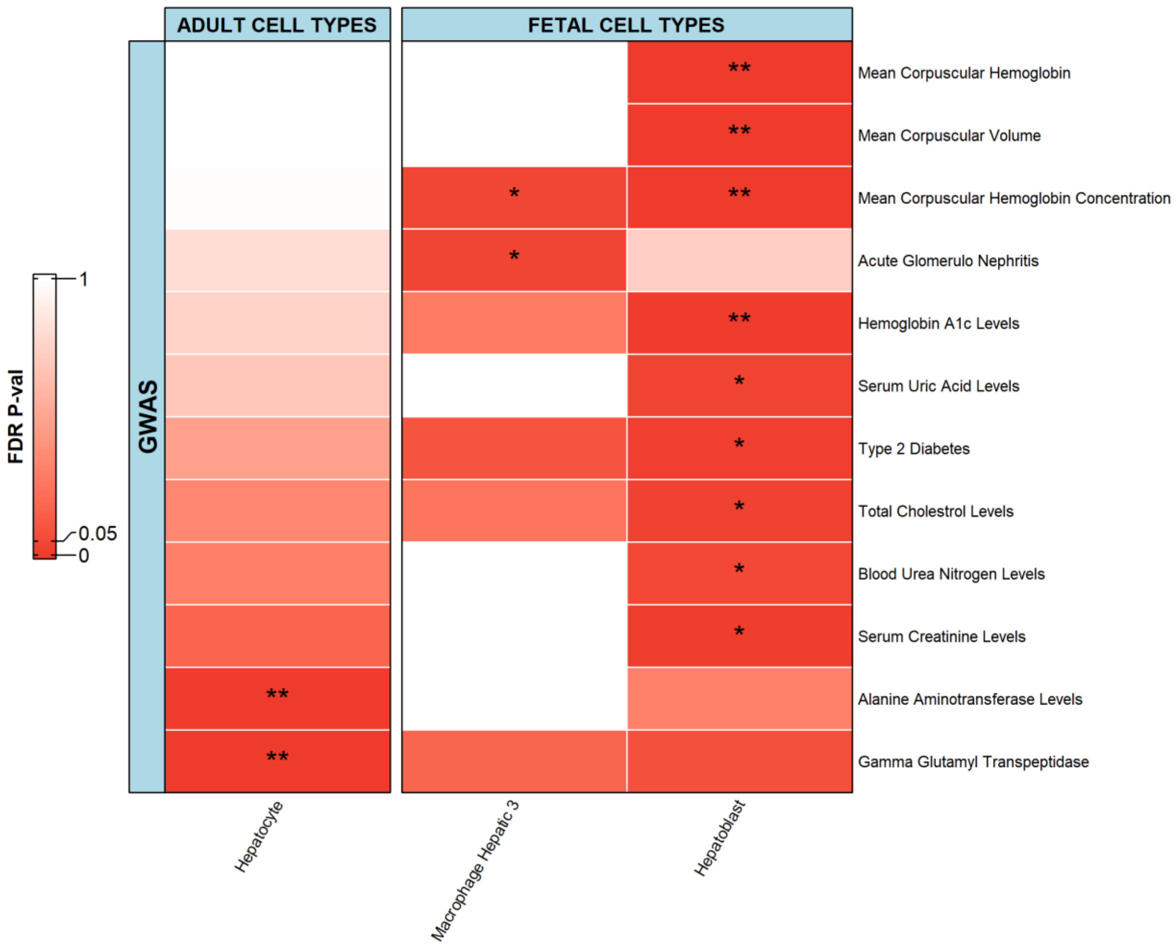


**Figure S11.** Heatmap depicting associations of hepatocytes from adult and fetal tissue cell types with respective phenotypes. The heatmap follows a gradient from red to white as the values go from 0.00 to 1.00. ** represents a significant association with an FDR adjusted p-value less than or equal to 0.01. * represents a significant association with an FDR adjusted p-value less than or equal to 0.05 but greater than 0.01.
