# Supplementary figures and images for: Single-Cell Chromatin Accessibility Data Combined with GWAS Improves Detection of Relevant Cell Types in 59 Complex Phenotypes
Source: Int J Mol Sci. 2022 Sep 28;23(19):11456. doi: 10.3390/ijms231911456 (PMC9570273; doi:10.3390/ijms231911456)

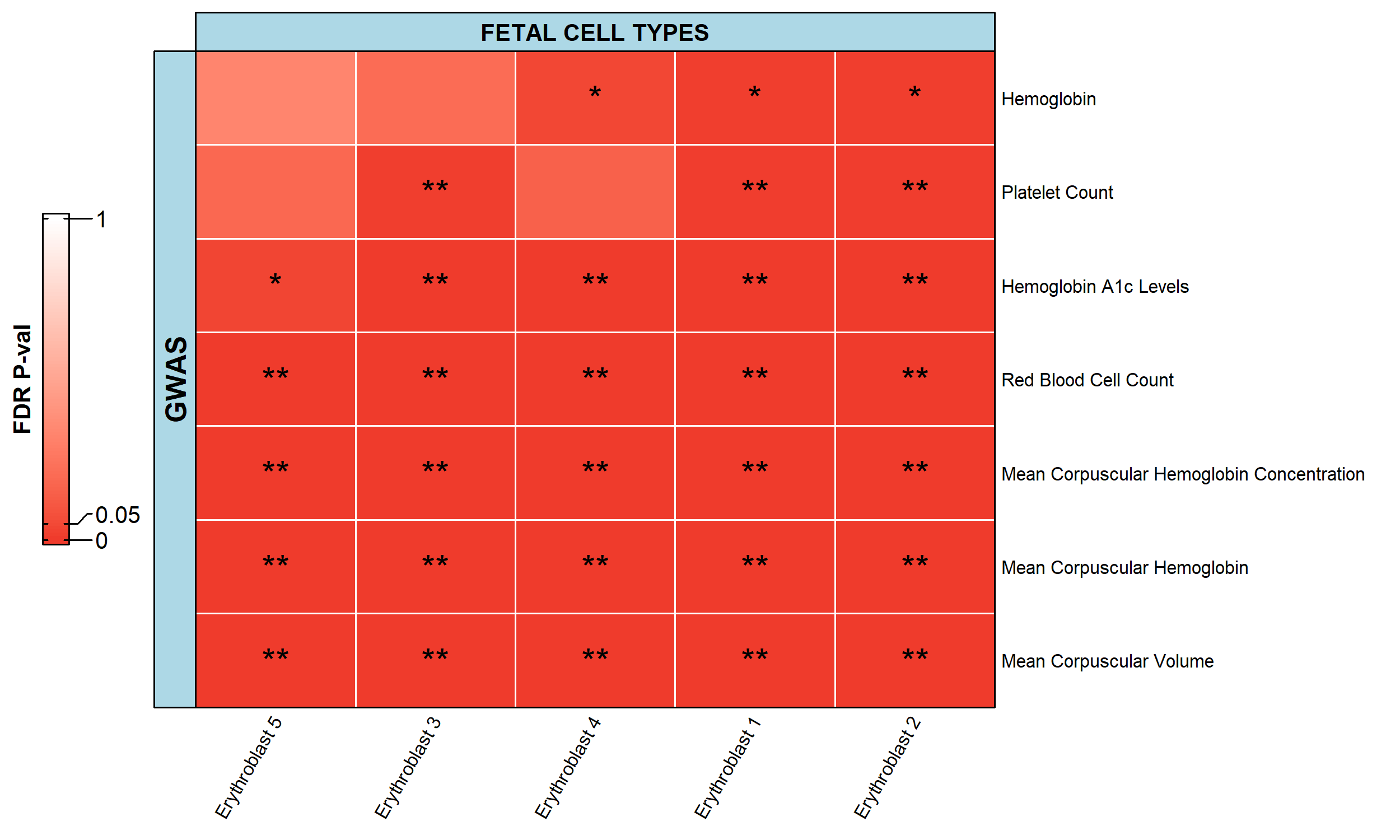

Supplement: Supplementary file 1 [file ijms-23-11456-s001.zip › FigureS1.png]

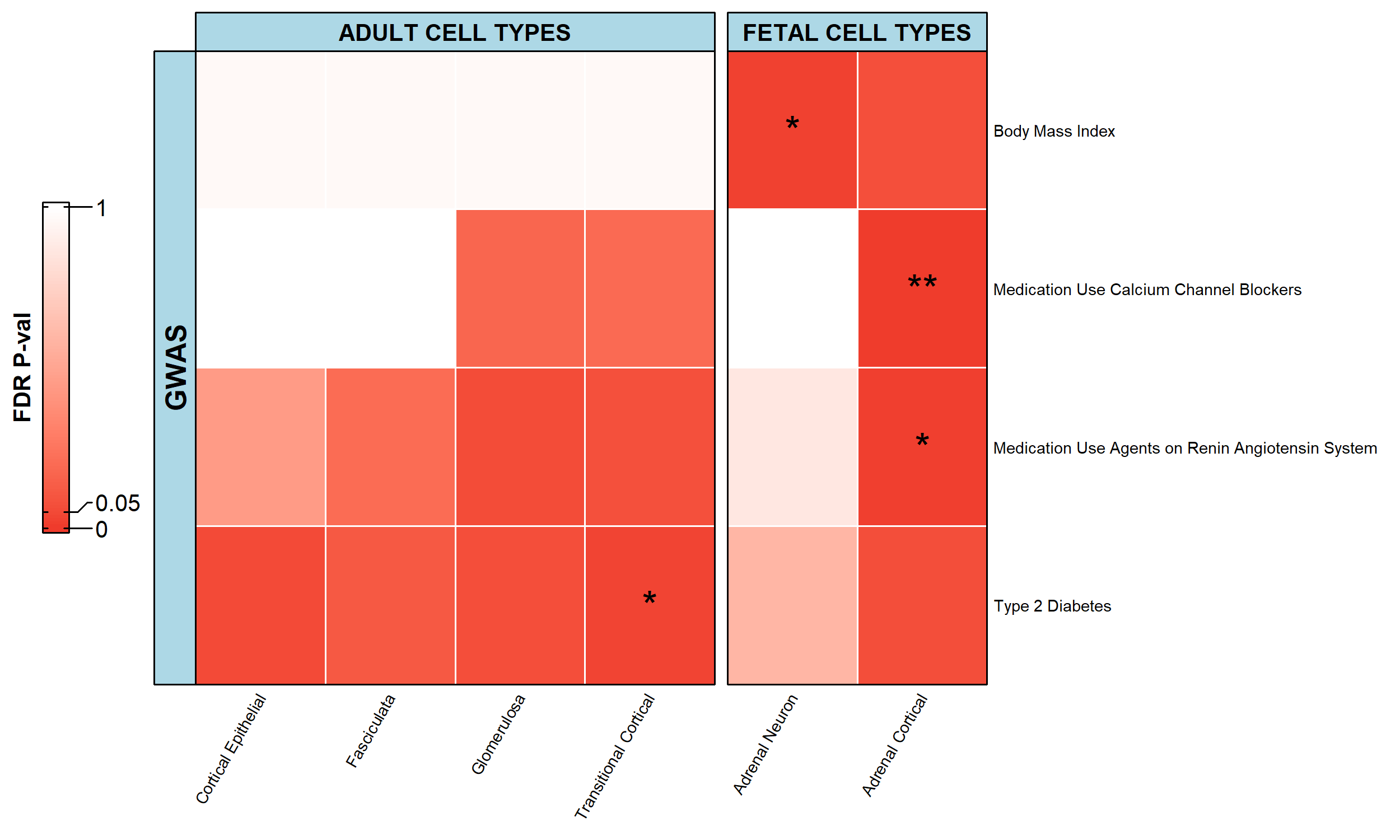

Supplement: Supplementary file 1 [file ijms-23-11456-s001.zip › FigureS10.png]

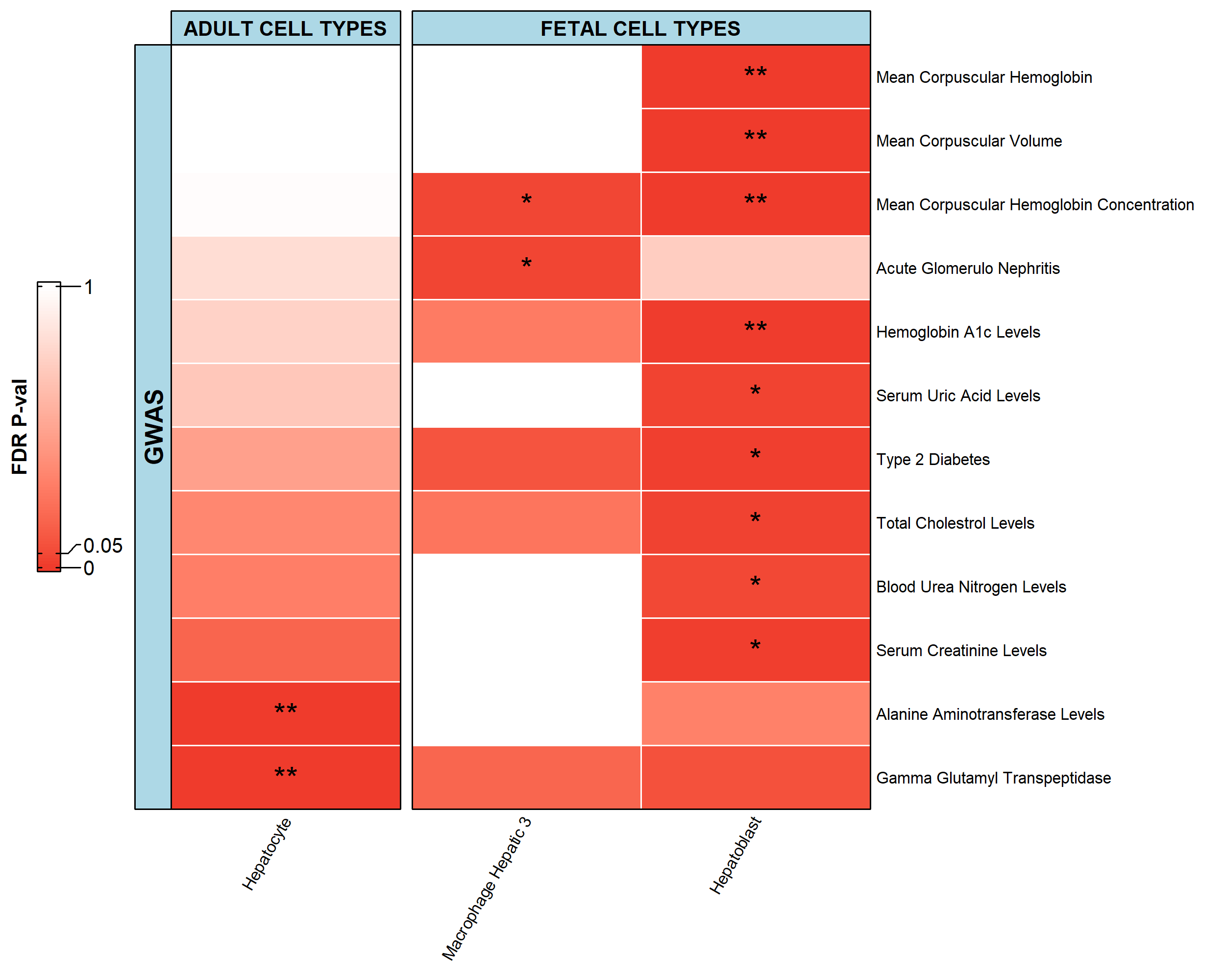

Supplement: Supplementary file 1 [file ijms-23-11456-s001.zip › FigureS11.png]

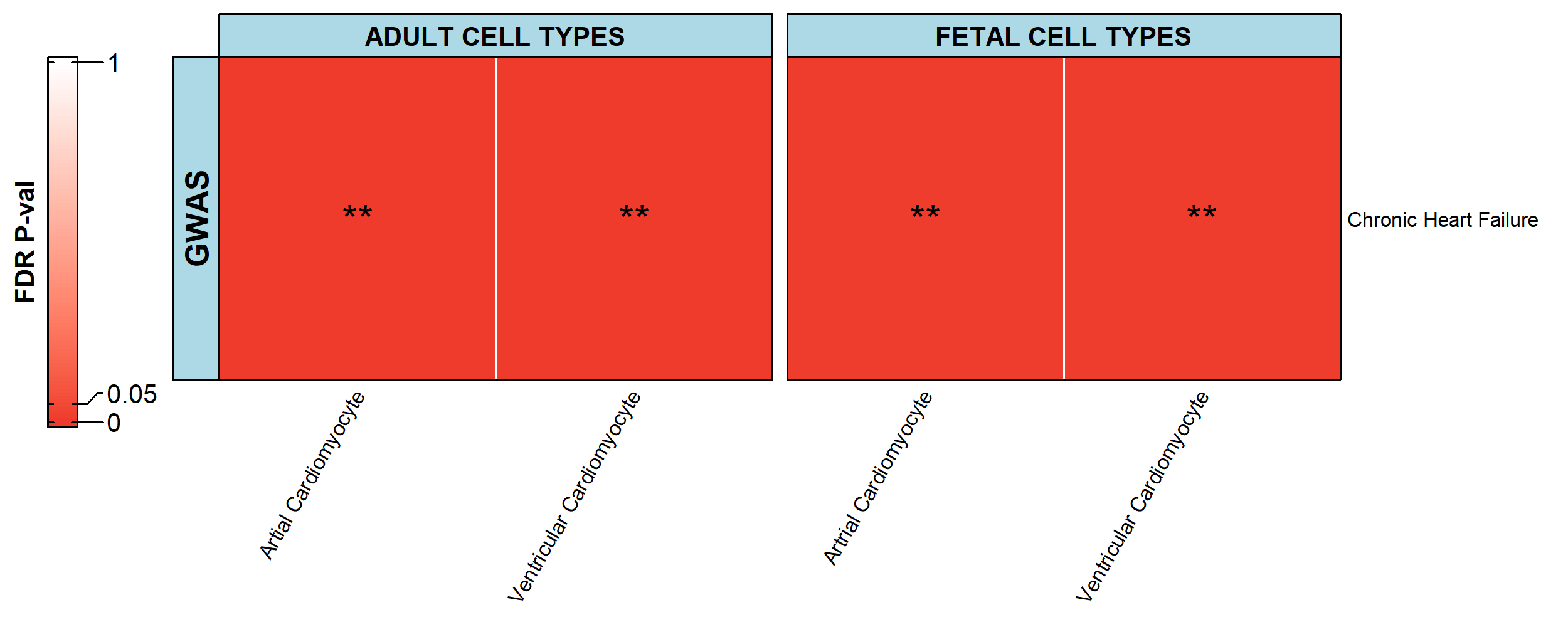

Supplement: Supplementary file 1 [file ijms-23-11456-s001.zip › FigureS2.png]

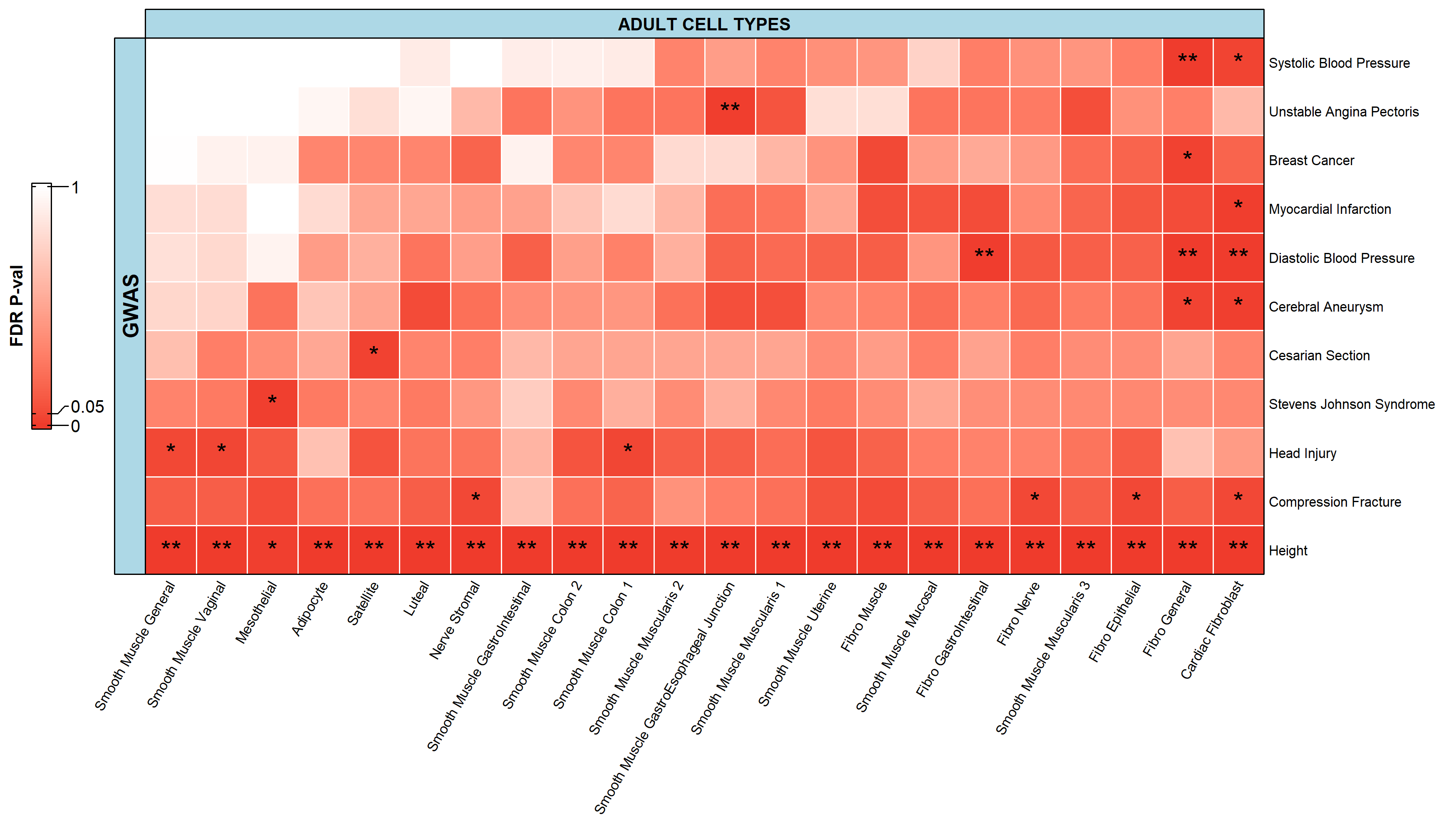

Supplement: Supplementary file 1 [file ijms-23-11456-s001.zip › FigureS3.png]

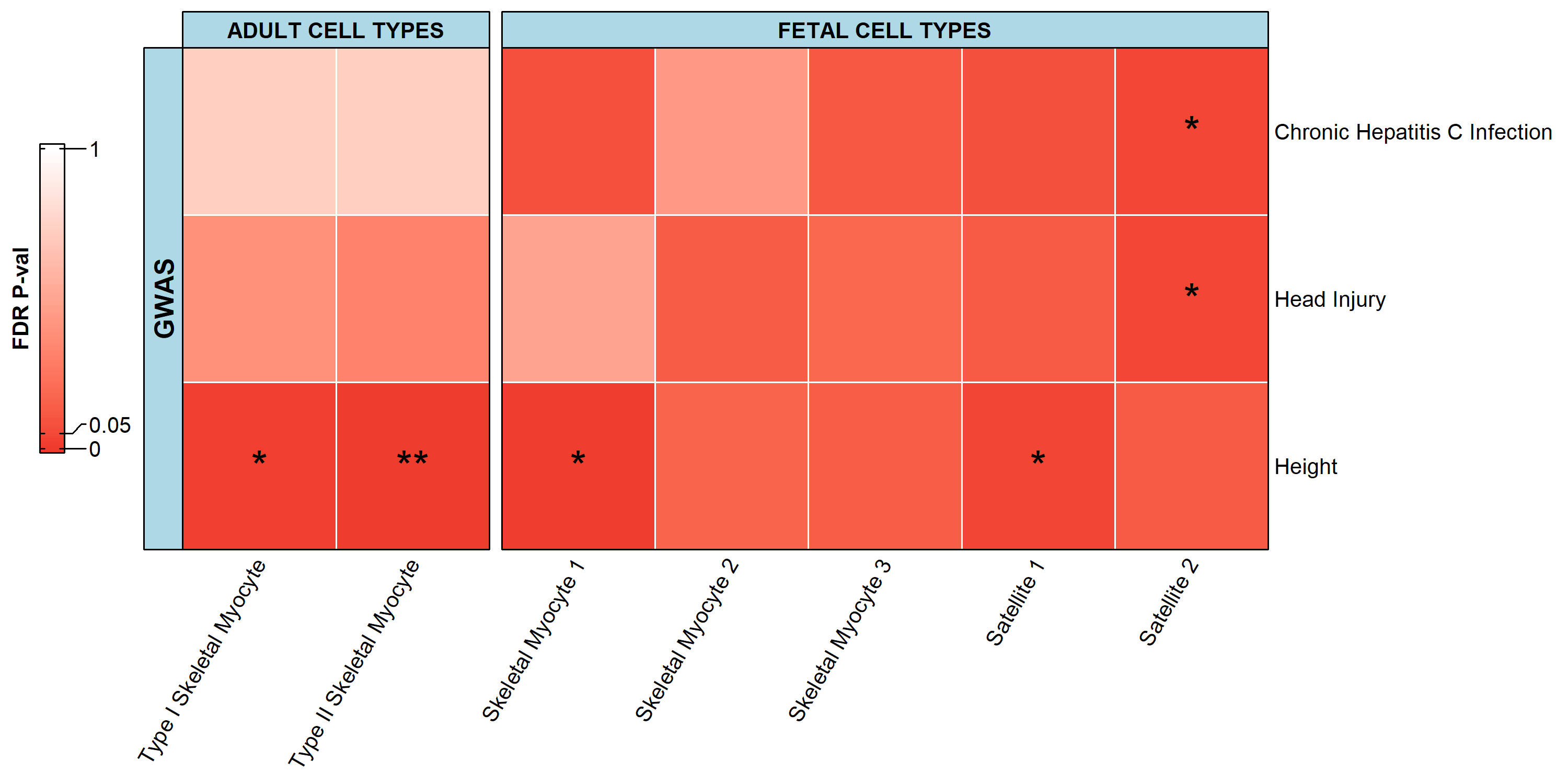

Supplement: Supplementary file 1 [file ijms-23-11456-s001.zip › FigureS4.png]

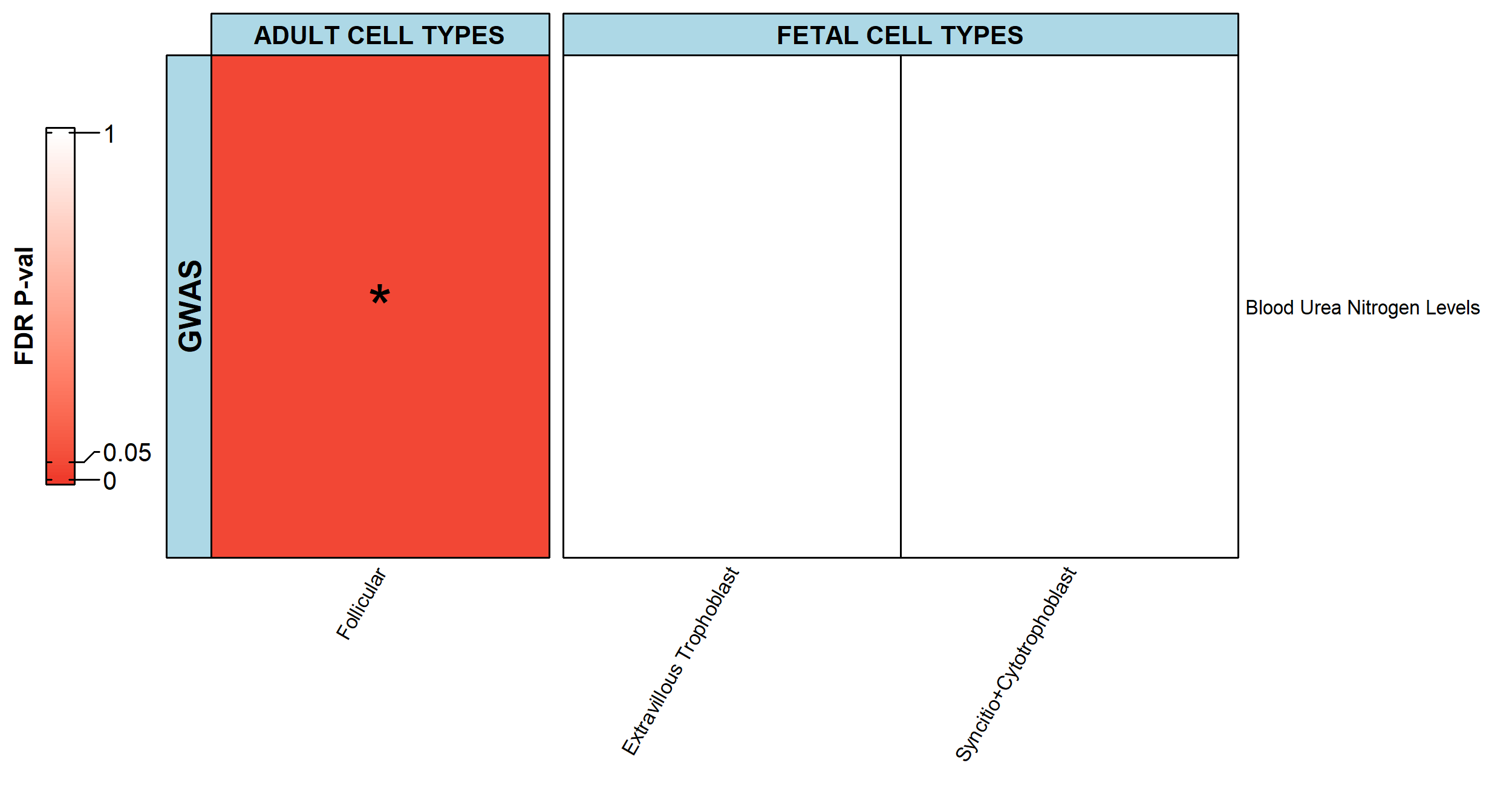

Supplement: Supplementary file 1 [file ijms-23-11456-s001.zip › FigureS5.png]

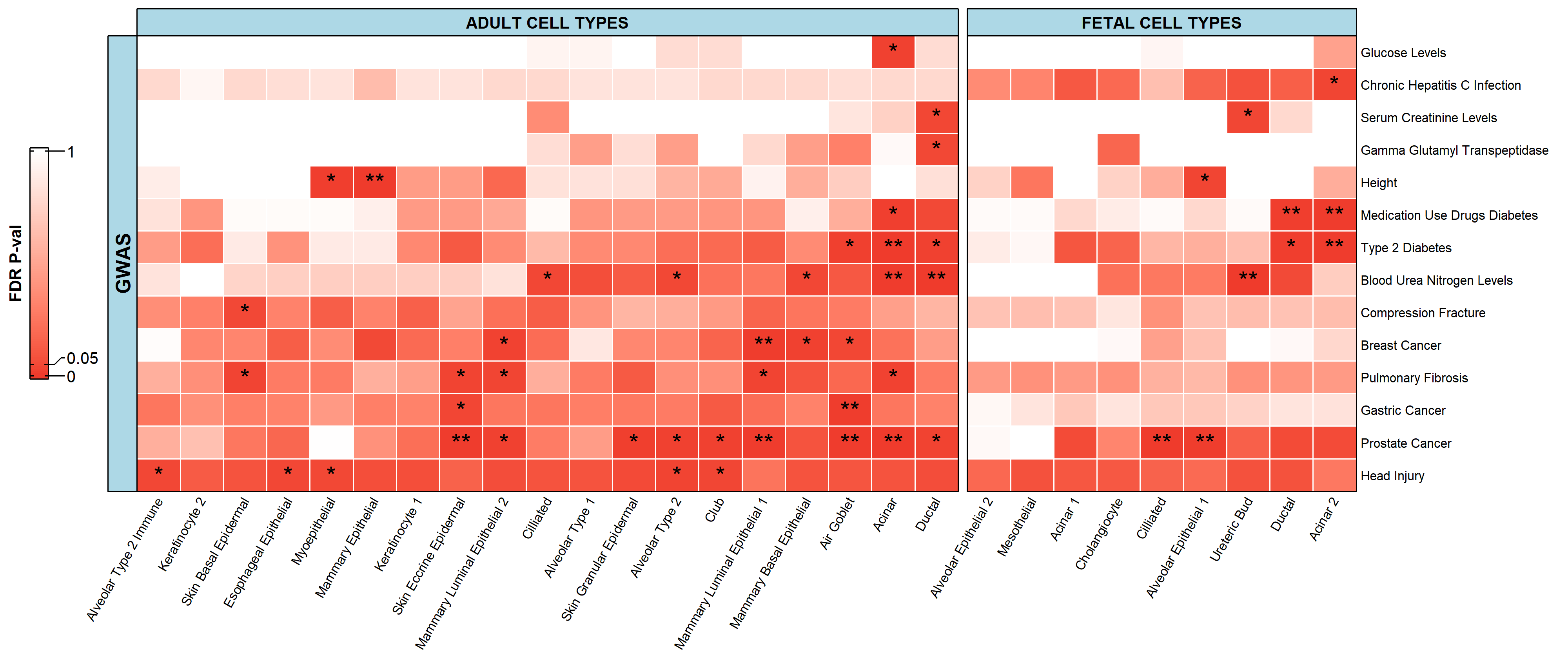

Supplement: Supplementary file 1 [file ijms-23-11456-s001.zip › FigureS6.png]

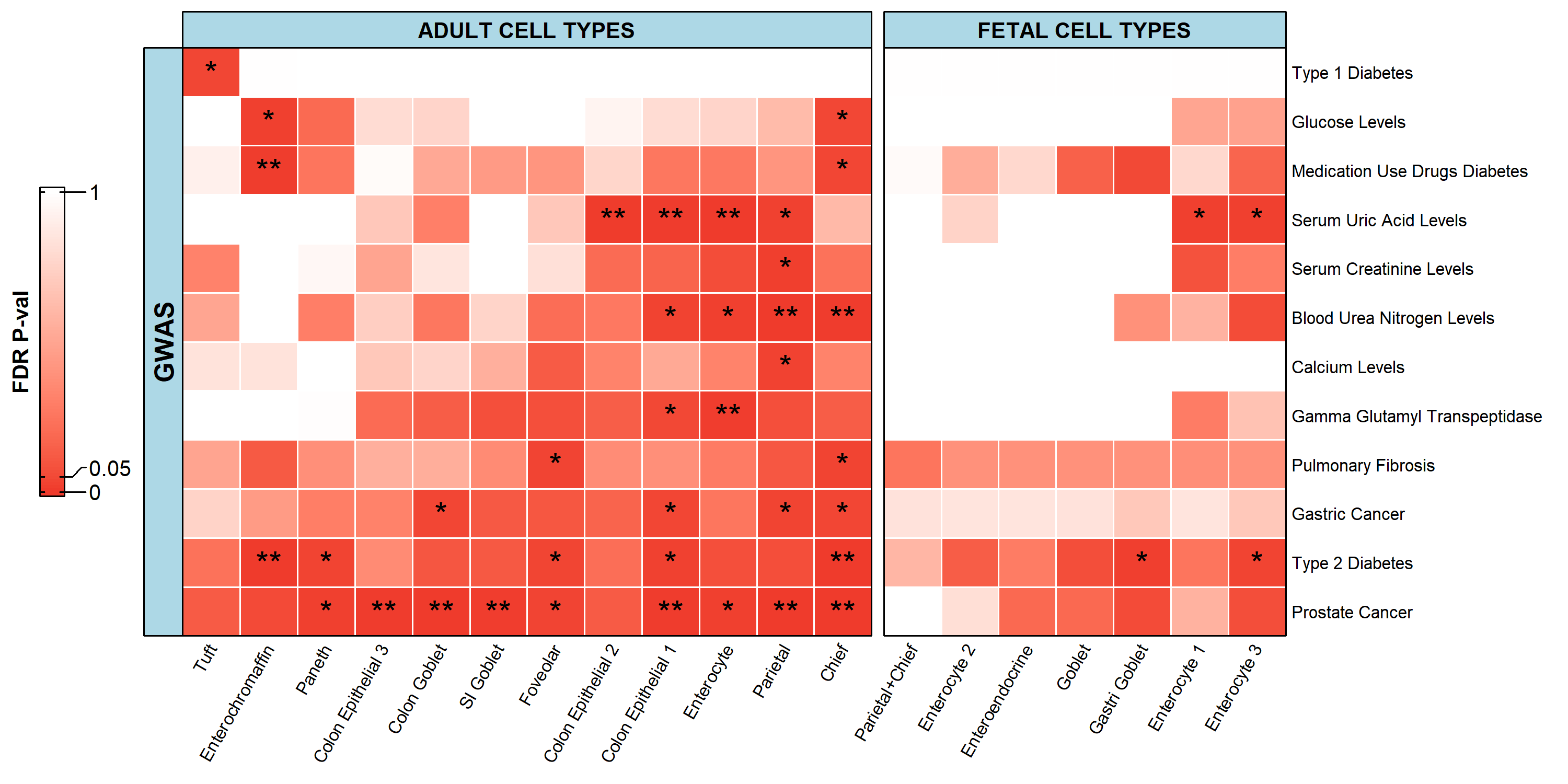

Supplement: Supplementary file 1 [file ijms-23-11456-s001.zip › FigureS7.png]

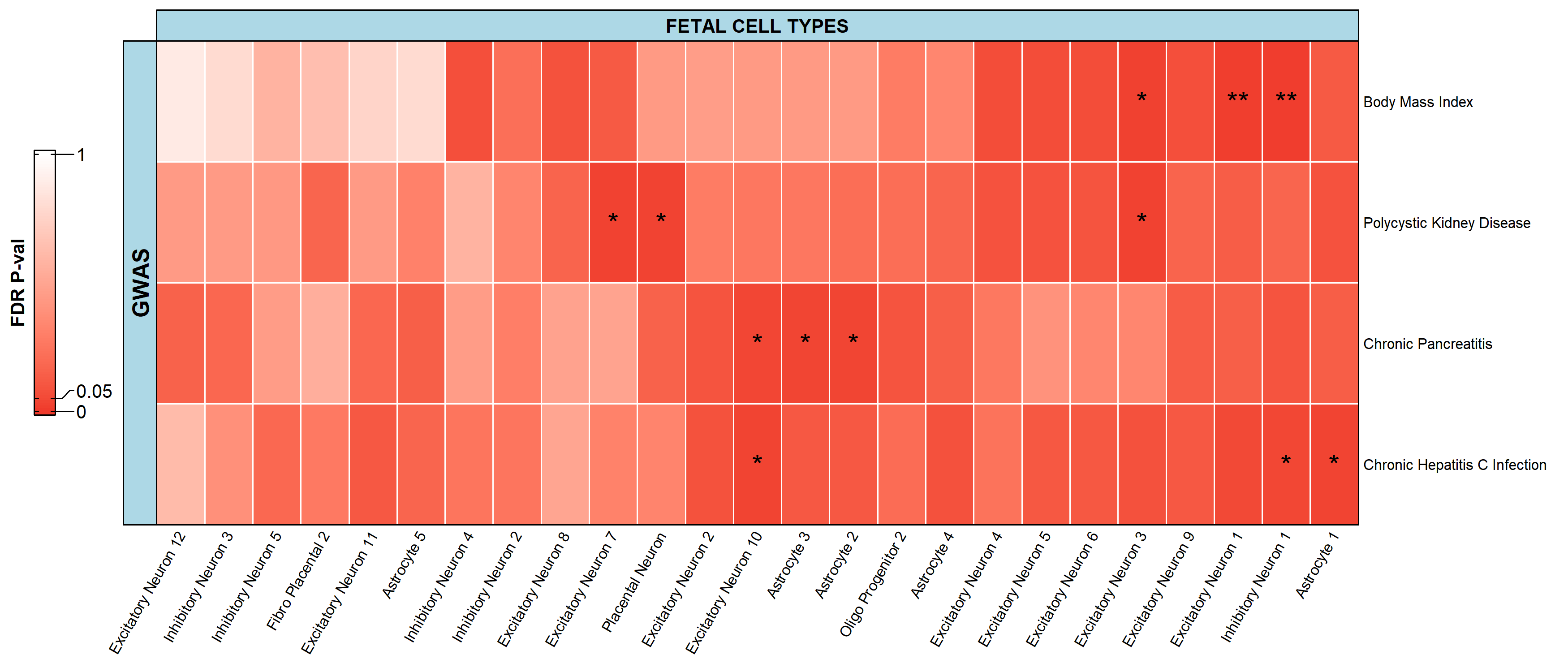

Supplement: Supplementary file 1 [file ijms-23-11456-s001.zip › FigureS8.png]

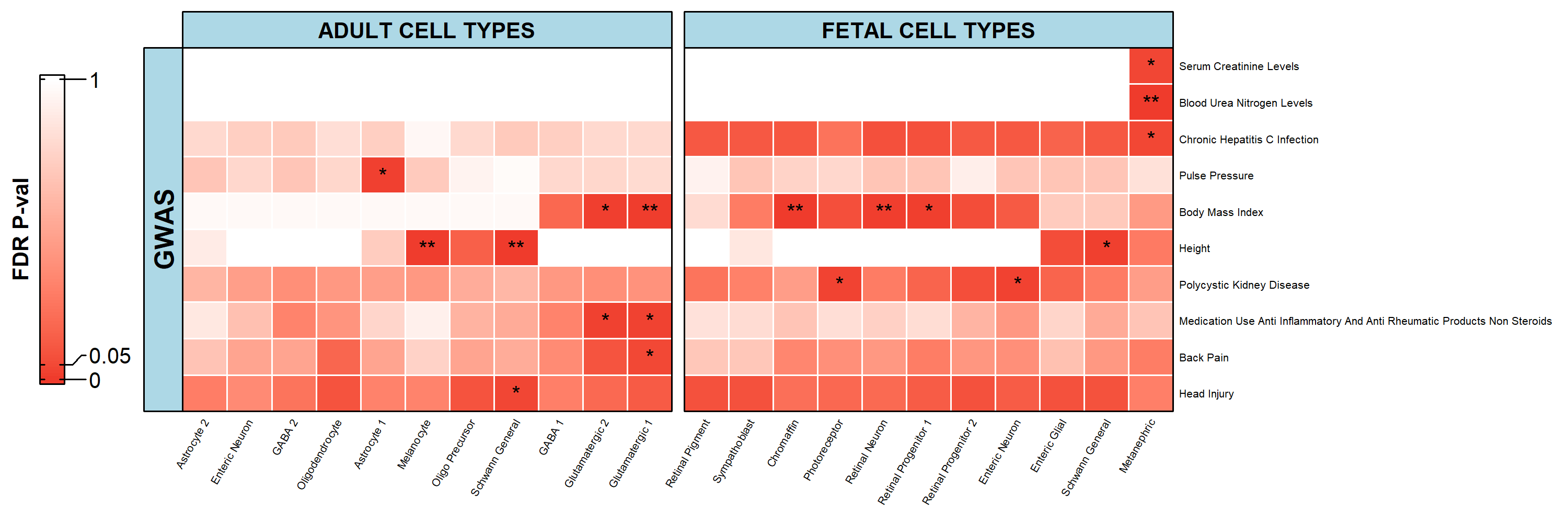

Supplement: Supplementary file 1 [file ijms-23-11456-s001.zip › FigureS9.png]
